# Diversity and evolution of four-domain voltage-gated cation channels of eukaryotes and their ancestral functional determinants

**DOI:** 10.1038/s41598-018-21897-7

**Published:** 2018-02-23

**Authors:** Ilya Pozdnyakov, Olga Matantseva, Sergei Skarlato

**Affiliations:** Institute of Cytology, Russian Academy of Sciences, Saint Petersburg, 194064 Russia

## Abstract

Four-domain voltage-gated cation channels (FVCCs) represent a large family of pseudo-tetrameric ion channels which includes voltage-gated calcium (Ca_v_) and sodium (Na_v_) channels, as well as their homologues. These transmembrane proteins are involved in a wide range of physiological processes, such as membrane excitability, rhythmical activity, intracellular signalling, etc. Information about actual diversity and phylogenetic relationships of FVCCs across the eukaryotic tree of life is scarce. We for the first time performed a taxonomically broad phylogenetic analysis of 277 FVCC sequences from a variety of eukaryotes and showed that many groups of eukaryotic organisms have their own clades of FVCCs. Moreover, the number of FVCC lineages in several groups of unicellular eukaryotes is comparable to that in animals. Based on the primary structure of FVCC sequences, we characterised their functional determinants (selectivity filter, voltage sensor, Na_v_-like inactivation gates, Ca_v_β-interaction motif, and calmodulin-binding region) and mapped them on the obtained phylogeny. This allowed uncovering of lineage-specific structural gains and losses in the course of FVCC evolution and identification of ancient structural features of these channels. Our results indicate that the ancestral FVCC was voltage-sensitive, possessed a Ca_v_-like selectivity filter, Na_v_-like inactivation gates, calmodulin-binding motifs and did not bear the structure for Ca_v_β-binding.

## Introduction

Four-domain voltage-gated cation channels (FVCCs) represent a family of ion channels of eukaryotes which belongs to the large and ancient voltage-gated cation channel superfamily^1^. FVCCs are known to be involved in a wide range of physiological processes, such as membrane excitability, rhythmical activity, intracellular signalling, etc^2^. At present, five genetic subgroups (subfamilies) of FVCCs are distinguished: 1) high voltage-activated calcium channels (HVA Ca_v_), i.e. Ca_v_1 and Ca_v_2; 2) low voltage-activated calcium channels (LVA Ca_v_), i.e. Ca_v_3; 3) voltage-gated sodium channels (Na_v_), i.e. Na_v_1 and Na_v_2 (Ca_v_4); 4) sodium leak channels (NALCN); and 5) calcium channels of fungi (Cch)^3,4^. It should be noted that characterised channels from all these subfamilies, except fungal Cch, are Metazoa-specific, and little is known about FVCCs of other eukaryotic groups. Considering the remarkable diversity of single-cell eukaryotes^5^, it is reasonable to assume that FVCCs are also very diverse and comprise more than five subfamilies.

For a long time, eukaryotic Na_v_ subfamily had been considered as a Metazoa-specific lineage of channels due to the role of sodium-selective channels in nervous system functioning. Surprisingly, recent studies showed that Na_v_ emerged before the split of eukaryotic supergroup Obazoa to Opisthokonta (the eukaryotic clade that unites metazoans, choanoflagellates, and fungi) and Apusomonadida^6^. At the same time, metazoan HVA and LVA Ca_v_ channels are thought to be more ancient than Na_v_, since four-domain voltage-gated calcium channels are present in eukaryotic lineages distant from Metazoa, such as green algae and ciliates^7^. However, there is no evidence of a close phylogenetic relationship between metazoan Ca_v_ and voltage-gated calcium channels of protists, with the only exception of FVCCs of choanoflagellates (the sister group to Metazoa) which are closely related to metazoan HVA Ca_v_^8^. Phylogenetic analysis of two other subfamilies of FVCCs – NALCN of metazoans and Cch of fungi – revealed that both subfamilies form a single clade of opisthokont voltage-insensitive channels^8^. It should be highlighted that although FVCC homologues seem to be abundant among different eukaryotic taxa, some groups of eukaryotes, such as Embryophytes (land plants) and Amoebozoa, lack FVCC genes^9,10^. Overall, the evolution of FVCCs and phylogenetic relationships between different lineages of this family are insufficiently understood. Moreover, it is not clear how well the currently defined five subfamilies of FVCCs cover their real diversity.

**Figure 1:**
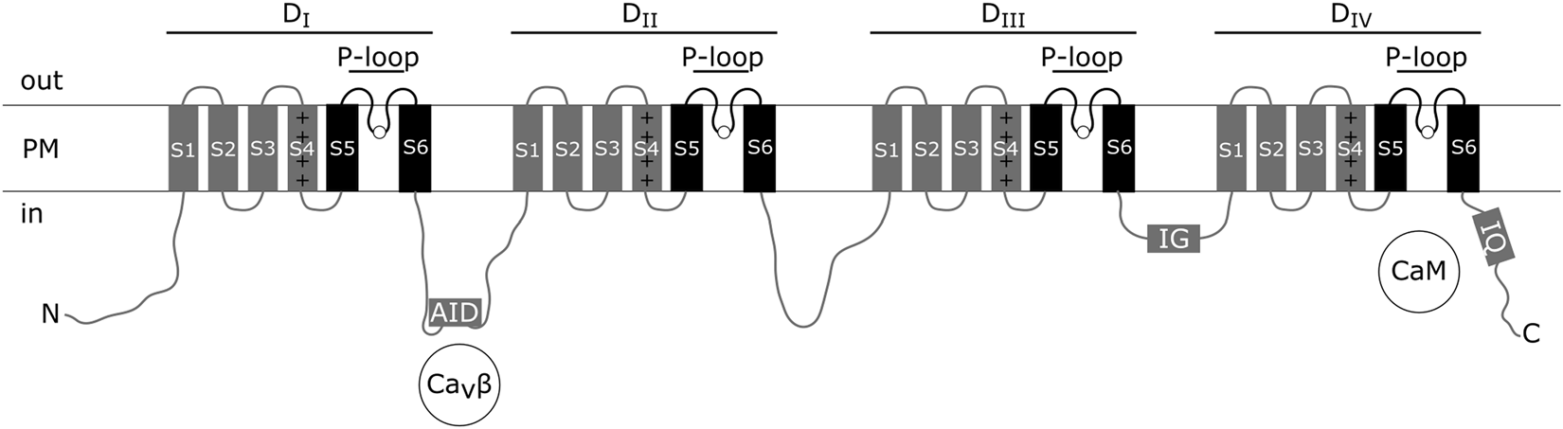
Schematic representation of the four-domain voltage-gated cation channel structure. Functional determinants considered in this work are shown. The pore-forming subunit consists of four homologous domains (D_I_–D_IV_). Each domain comprises six transmembrane segments (S1–S6). Segments S4 may bear positive charges (+) and function as a voltage sensor. Segments S5 and S6 together with a pore-loop (P-loop) form a pore of a channel. Four amino acid residues, one from each P-loop (white circles), form a selectivity filter. AID – α-interaction domain; CaM – calmodulin; Ca_v_β – auxiliary β-subunit of HVA Ca_v_; IG – Na_v_-type inactivation gates; IQ – calmodulin-binding region containing pre-IQ and IQ motifs; PM – plasma membrane.

All FVCCs have a similar pseudo-tetrameric architecture^2,3^ (Fig. 1). The pore-forming subunit of FVCC consists of four homologous domains (D_I_–D_IV_) in a single polypeptide chain. Each domain comprises six transmembrane segments (S1–S6). Many structures of FVCCs, such as segments S4, selectivity filter, inactivation gates, etc., are critical determinants of their functional activity, but the order of acquisition and evolution of these structures still have to be elucidated.

Segments S4 are often rich in positively charged amino acid residues – arginine and lysine, which is essential to their functioning as a voltage sensor^2^. Three known voltage-insensitive groups of FVCCs, i.e. metazoan NALCN, fungal Cch, and a subgroup of mammalian Na_v_1 channels - Na_x_, possess arginine/lysine-poor segments S4^11–13^, i.e. S4s that bear less than three positive charges or S4s with positive charges separated by an extended sequence of hydrophobic amino acid residues.

Segments S5, S6 and a ‘pore-loop’ (P-loop) between them from each of four domains compose the entire pore of FVCC. Four amino acid residues in a pore (one from each of four P-loops) form a selectivity filter playing a crucial role in ion selectivity of a channel^2^. Many studies confirm the importance of lysine (K) for sodium selectivity or at least permeability of FVCCs (selectivity filters D/E/K/A or D/K/E/A in sodium-selective Na_v_, E/K/E/E or E/E/K/E in sodium-permeable NALCN)^14,15^. So far characterised FVCCs without lysine (K) in a selectivity filter represent calcium-selective (selectivity filters E/E/E/E in HVA Ca_v_; E/E/D/D in LVA Ca_v_) or even cation non-selective channels (selectivity filters D/E/E/A in Na_v_; E/E/E/E in NALCN).

One-domain prokaryotic voltage-gated sodium channels, often referred to as BacNa_v_, represent a tetramer consisting of four subunits homologous to a single domain of FVCCs. Surprisingly, a selectivity filter motif of these sodium-selective channels is E/E/E/E. However, although both eukaryotic FVCCs and prokaryotic BacNa_v_ channels evolved from a common ancestral one-domain voltage-gated potassium channel, these channel families represent two distantly related evolutionary lineages of the voltage-gated cation channel superfamily. Sodium-selectivity was acquired in these channel families independently and is likely underlain by different structural traits including not only a selectivity filter itself but also its surroundings^16^. It is already known that besides a selectivity filter, other P-loop sites influence the selectivity of FVCCs. Amino acid residues in the positions n + 1 relative to the selectivity filter residues (i.e. amino acid residues following each of four residues comprising a selectivity filter motif) contribute to the calcium selectivity of both eukaryotic FVCCs and prokaryotic BacNa_v_. Indeed, HAV Ca_v_ and LVA Ca_v_ channels possess aspartate in the n + 1 position of D_II_, whereas sodium-selective Na_v_ channels do not bear a negatively charged residue in this position^17,18^.

An important feature of metazoan Na_v_ channels is a specific mechanism of fast inactivation ensured by the structure called ‘inactivation gates’^19^. Inactivation gates represent a motif of three hydrophobic (Φ) and one polar non-charged amino acids (threonine (T) or serine (S)) residues, ΦΦΦT(S), which is situated in the intracellular loop between D_III_ and D_IV_. Fast inactivation plays an essential role in Na_v_ channel functioning and nervous signal propagation in metazoans. Hence, for a long time, inactivation gates motif was considered as an exclusive structure of Na_v_. Recently homologous structures have been found in FVCC sequences of several protistan species^20^.

In Na_v_ channels, the process of fast inactivation involves an interaction of inactivation gates and a docking site. The structure of the docking site is not fully deciphered. Site-directed mutagenesis studies demonstrated that several channel structures take part in the formation of the docking site: S4-S5 intracellular loops of D_III_ (alanine 1329 in rat Na_v_1.2) and D_IV_ (phenylalanine 1651 in rat Na_v_1.2), as well as S5 of D_IV_ (asparagine 1662 in rat Na_v_1.2)^21,22^. Recent data on the structure of Na_v_1.4 of the electric eel confirm that these amino acid residues interact with inactivation gates^23^.

HVA Ca_v_ and LVA Ca_v_ channels do not possess inactivation gates motif and are controlled by other mechanisms of inactivation. In HVA Ca_v_, inactivation is regulated by means of an auxiliary cytosolic β-subunit (Ca_v_β) which interacts with the ‘α-interaction domain’ (AID) situated in the intracellular loop between D_I_ and D_II_^24^. LVA Ca_v_ do not possess AID and therefore do not interact with Ca_v_β^25^. Activation and inactivation of metazoan Na_v_ and HVA Ca_v_ channels are also controlled through interaction with calmodulin^26^. In this process, calmodulin binds to conservative C-terminal pre-IQ and IQ motifs of these channels^27^.

Previously it was demonstrated that some nonopisthokont FVCCs possess an HVA Ca_v_-like selectivity filter and a Na_v_-like inactivation gates motif in a single channel^20^. This unexpected conjunction of functional determinants suggests intricate FVCC evolution and indicates that yet uncharacterised FVCCs of unicellular eukaryotes may have unusual properties. The aim of this work was to identify homologues of metazoan and fungal FVCCs in a broad spectrum of eukaryotic organisms and investigate their phylogeny and structural diversity in order to shed light on the evolution of these channels.

## Results

### Phylogeny of FVCCs

**Figure 2:**
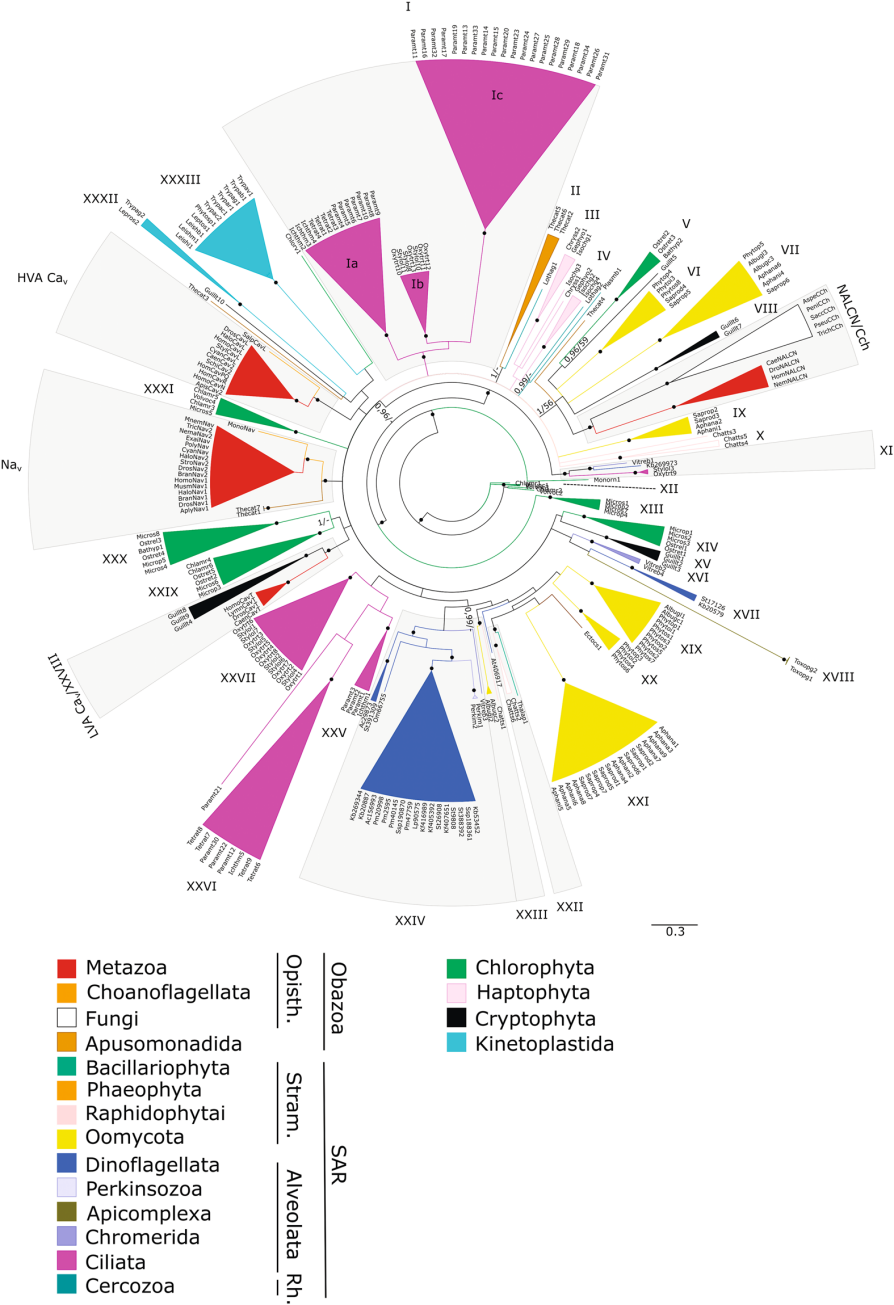
Collapsed phylogenetic tree of eukaryotic four-domain voltage-gated cation channels. Different colours correspond to different eukaryotic groups. Black circles indicate nodes with posterior probability/bootstrap value ≥ 0.95/ ≥ 65 (well-supported clades (WSCs)). Every WSC, except voltage-gated sodium channels (Na_v_), high-voltage activated calcium channels (HVA Ca_v_), low-voltage activated calcium channels (LVA Ca_v_), and sodium leak channels/calcium channels of fungi (NALCN/Cch), has been assigned a number (I–XXXIII). WSCs containing channels from more than one of 18 eukaryotic lineages are shaded. Opisth. – Opisthokonta; Stram. – Stramenopiles; Rh. – Rhizaria.

**Figure 3:**
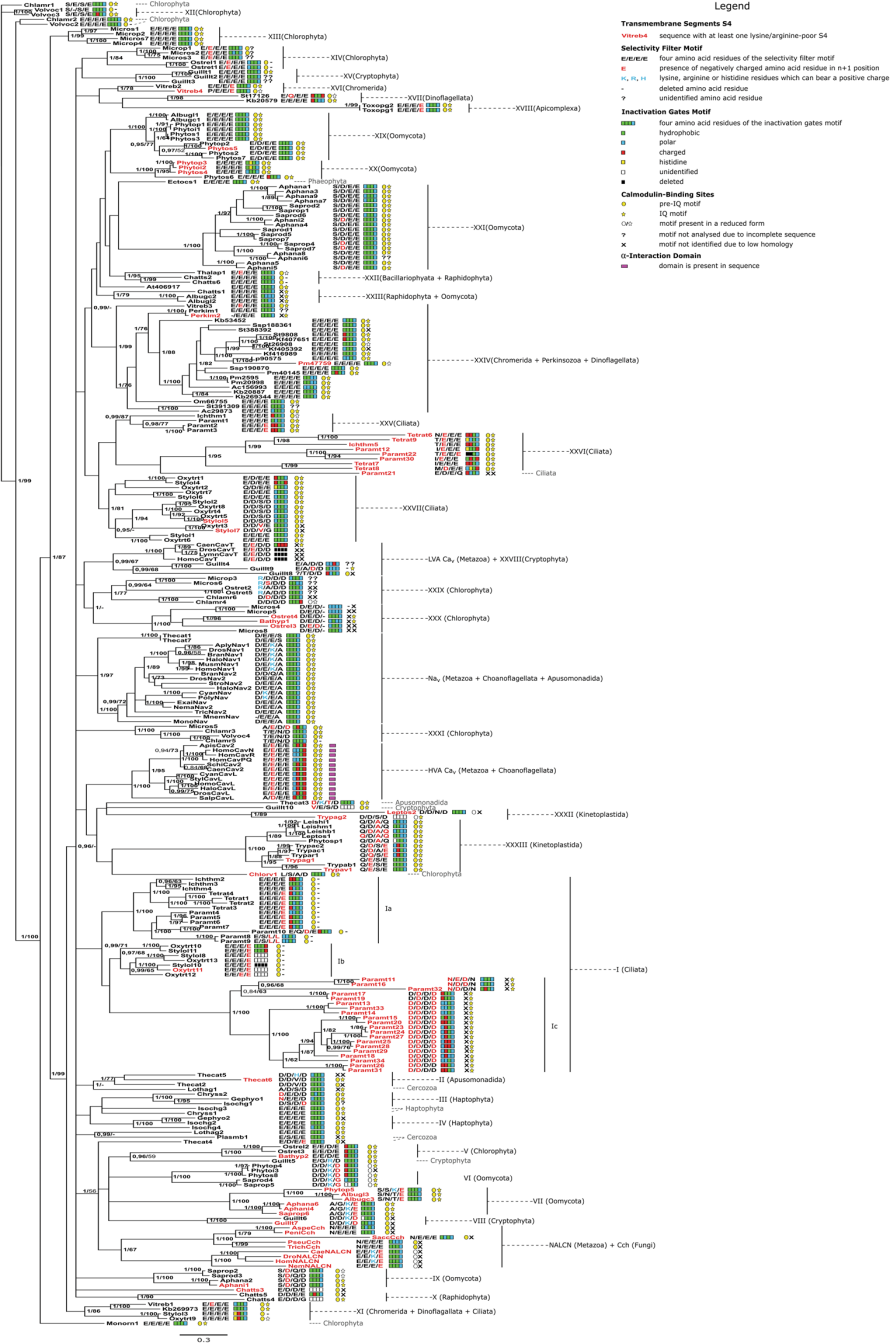
Functional determinants of four-domain voltage-gated cation channels of eukaryotes mapped on their full Bayesian phylogenetic tree.Bootstrap values < 65 and posterior probabilities <0.95 are not shown.

In this work, we performed the taxonomically broad Bayesian and maximal likelihood phylogenetic analyses (Fig. 2, Supplementary Fig. S1) of 277 FVCCs from different groups of eukaryotes (Supplementary Table S1) and mapped their predicted structural features on the obtained trees (Fig. 3). The dataset included protein sequences of four-domain channels of Metazoa (Na_v_, HVA Cav, LVA Cav, and NALCN), Fungi (Cch), and protists (predominantly FVCCs with uncharacterised properties).

Both phylogenetic trees inferred by Bayesian and maximal likelihood methods are in agreement with each other. However, these trees are unresolved in many deep nodes, and the order of divergence among distinct clades of FVCCs, including metazoan Na_v_ and Ca_v_ channels, cannot be deciphered (Fig. 2). Nevertheless, phylogenetic analysis showed that actual diversity of FVCCs is very complex and not limited to five conventional genetic subfamilies. All 277 sequences form 36 well-supported clades (WSC, posterior probability ≥0.95, bootstrap value ≥ 65%) and 18 distinct lone branches. The results indicate that many groups of eukaryotes possess their own clades of four-domain channels which do not cluster with Na_v_, HVA Ca_v_, LVA Ca_v_, NALCN, or Cch. Moreover, the number of FVCC lineages in some groups of unicellular eukaryotic organisms, such as dinoflagellates (WSCs XI, XVII, XXIV, At406917) and oomycetes (WSCs VI, VII, XIX–XXI, XXIII), is not fewer than that in multicellular animals.

### Structural diversity of FVCCs

The high phylogenetic diversity of FVCCs may reflect the structural and functional diversity of these channels. Therefore, we analysed the primary structure of several functional determinants, i.e. selectivity filter motif, transmembrane segments S4, region homologous to inactivation gates motif of Na_v_, region homologous to AID of HVA Ca_v_, and pre-IQ/IQ motifs, in all 277 protein sequences of FVCCs and then mapped them on the FVCC phylogeny.

#### Selectivity filter

Analysis of the primary structure of P-loop regions revealed a great variety of selectivity filter motifs characteristic to FVCCs of different eukaryotes. Nevertheless, E/E/E/E motif (four glutamate residues) is dominant and appears in most clades of four-domain channels and in evolutionary distant groups of organisms, such as metazoans, chlorophytes, oomycetes, dinoflagellates, haptophytes, etc. (Fig. 3).

Among other identified selectivity filter motifs, several types attract special attention. Some FVCC sequences of oomycetes and cryptophytes possess lysine-containing selectivity filters (Fig. 3). Specifically, sequences from WSC VI (*Phytophthora* and *Saprolegnia*, Oomycota) and WSC VIII (*Guillardia*, Cryptophyta) possess selectivity filters D/D/K/D(G) which resemble the selectivity filter E/E/K/E of sodium-permeable NALCN (one positively charged lysine (K) among three negatively charged amino acid residues – either aspartic acid (D) or glutamic acid (E)). Three residues of the other lysine-containing selectivity filter motif A/G/K/E found in oomycetes from WSC VII (*Aphanomyces* and *Saprolegnia*) are identical to three residues of the classical D/E/K/A filter of Na_v_. One sequence of the oomycete *Phytophthora* (WSC VII) and one sequence of the apusomonad *Thecamonas* have unusual selectivity filter motifs S/S/K/E and D/K/T/D, respectively, in which lysine (K) is surrounded by negatively charged residues of glutamic (E) or aspartic (D) acids and polar residues of serine (S) or threonine (T).

Notably, selectivity filters with positively charged residues are rare across our dataset. Apart from metazoan Na_v_ and NALCN, as well as already mentioned FVCCs of oomycetes, a cryptophyte, and an apusomonad, we found such selectivity filters in four sequences of *Micromonas* and *Ostreococcus* (Chlorophyta, Prasinophyceae; WSC XXIX), *Guillardia* (Cryptophyte), and *Thecamonas* (Apusomonadida; WSC II). However, selectivity filters of these sequences are unique, because they contain arginine (R) or histidine (H) instead of lysine (K) as a positively charged residue (Fig. 3).

The analysis showed that the n + 1 positions are less conservative than a selectivity filter motif. The presence of negatively charged amino acid residues in n + 1, known to be important for the calcium selectivity, does not correlate with the presence of E/E/E/E motif in the FVCC sequences (Fig. 3). Not all E/E/E/E-containing FVCCs possess D or E in the positions n + 1, and conversely, negatively charged n + 1 amino acid residues are not exclusively attributed to the channels containing E/E/E/E selectivity filter. Similar to NALCN, all FVCCs of oomycetes, cryptophytes, and apusomonads containing lysine in their selectivity filter possess D or E in the n + 1 position of D_IV_ (oomycetes and cryptophytes) or D_I_ and D_III_ (apusomonads).

#### Segments S4

In all characterised FVCCs, arginine/lysine-rich transmembrane segments S4 serve as voltage-sensors. We analysed arginine/lysine content in S4 segments of non-characterised protistan channels. The analysis showed that FVCCs with all four arginine/lysine-rich S4s are dominant and can be found in evolutionary distant groups of eukaryotes (Fig. 3, Supplementary Table S2). Nevertheless, some clades of protistan FVCCs exclusively or predominantly consist of channels with at least one arginine/lysine-poor S4 (e.g. subclade Ic and WSC XXVI of ciliates). At the same time, there are clades of protistan FVCCs representing a mixture of channels with arginine/lysine-rich S4s and channels with at least one arginine/lysine-poor S4 (e.g. WSC XXXIII of kinetoplastids) (Fig. 3, Supplementary Table S2). A bunch of sequences bears considerable modifications of S4, ranging from partial (e.g. sequence Pm47759 (*Prorocentrum minimum*, Dinoflagellata)) or complete (e.g. sequence Stylol5 (*Stylonychia lemnae*, Ciliata)) deletion of S4 to insertions separating positive charges in this region (e.g. sequences Paramt11–Paramt34 (*Paramecium tetraurelia*, Ciliata)) (Supplementary Table S2). Notably, many sequences of unicellular eukaryotes possess S4s containing residues of histidine which can be positively charged at pH ≤ 7.

#### Determinants of activity regulation: inactivation gates, α-interaction domain (AID), and pre-IQ/IQ motifs

In order to study diversity and distribution of motifs homologous to inactivation gates among eukaryotic FVCCs, we analysed the primary structure of the intracellular loop between D_III_ and D_IV_ (Fig. 3, Supplementary Table S3). We found that the canonical structure of Na_v_-like inactivation gates (motif ΦΦΦT(S)) is abundant among different clades of FVCCs from evolutionary distant groups of organisms. At the same time, many FVCC sequences bear substitutions in this functional determinant. For example, polar or charged residues substitute hydrophobic ones in the ΦΦΦ triplet of the inactivation gates structure in FVCCs from WSC XXVI (Ciliata) and HVA Ca_v_.

We also examined all sequences for the potential presence of the inactivation gates docking site (Supplementary Table S3). Based on the literature data, positions corresponding to alanine (A) 1329, phenylalanine (F) 1651, and asparagine (N) 1662 in the rat Na_v_1.2, were chosen as crucial to the inactivation gates docking. We found that asparagine in that position is a very conservative feature characteristic to most of the analysed sequences. Alanine appeares to be less common but is still widely distributed among FVCCs from different groups of eukaryotes. Finally, the position corresponding to phenylalanine 1651 in the rat Na_v_1.2 contains diverse amino acid residues in different FVCC sequences. The complete A/F/N motif was found only in metazoan Na_v_ channels. However, the A/X/N motif is rather conservative and can be found in FVCCs of evolutionary distant eukaryotic organisms. Remarkably, the presence of this motif is not exclusively attributed to ΦΦΦT(S)-containing sequences (Supplementary Table S3).

Analysis of the intracellular loop between D_I_ and D_II_ demonstrated that AID responsible for interaction of HVA Ca_v_ with Ca_v_β is exclusively present in the sequences from HVA Ca_v_ clade (Fig. 3).

We analysed the primary structure of regions homologous to the most conservative pre-IQ and IQ sequences (12 and 11 amino acid residues, respectively) (Fig. 3, Supplementary Table S3). Overall, motifs homologous to pre-IQ and IQ are widely spread among various clades of the FVCC phylogenetic tree. However, in some FVCC sequences, these motifs are eliminated or partly reduced. Evolution of both calmodulin-binding motifs (pre-IQ and IQ) seems to be independent because some FVCCs bear only one of the two motifs.

Overall, most FVCC clades are characterised by the dominant pattern of functional determinants (Fig. 3). A bright example of such dominant patterns can be found in WSC I unifying FVCC sequences of ciliates. This clade consists of three subclades, Ia, Ib, and Ic. Ia and Ib are characterised by a combination of the arginine/lysine-rich S4s, dominant selectivity filter E/E/E/E, pre-IQ motif, and deletion of the IQ motif. Ic unites FVCCs with at least one arginine/lysine-poor S4, the dominant selectivity filter D/D/D/D, and IQ motif. The pre-IQ motif is undetectable in the Ic sequences due to low sequence homology of the respective region. FVCCs from Ia and Ic are characterised by accumulation of polar and charged amino acid residues in the inactivation gates region, whereas this motif is absent or undetectable in most FVCCs of Ib.

## Discussion

Due to historical and methodological reasons, most FVCCs characterised to date belong to animals and fungi and only a few – to unicellular organisms from other eukaryotic lineages. Therefore, it is quite natural that five presently distinguished subfamilies of FVCCs unify metazoan and fungal channels. However, our phylogenetic analysis shows that homologues of FVCCs are widely spread in genomes and transcriptomes of organisms from all major eukaryotic groups and form their own subfamilies, distinct from Na_v_, HVA Ca_v_, LVA Ca_v_, NALCN, and Cch. This suggests the rich history of FVCC family, but the order of evolutionary events is still enigmatic because the obtained phylogeny is unresolved in its deep nodes. The reason for that may lie in both the high degree of FVCC variability and in the high rate of early eukaryotic evolution. The latter is also a probable reason for the unresolved phylogeny of eukaryotes^5^.

Remarkable variety of protistan FVCCs raises questions concerning their role in the physiology of unicellular eukaryotes. In well-studied animals, these proteins are involved in many essential processes, from action potential propagation to rhythmical activity of certain cell types. Notably, the existence of ion channels of Na_v_, HVA Ca_v_, LVA Ca_v_ and NALCN subfamilies with different functional traits underpins sophisticated cell signalling typical for animals. Some groups of protists, such as oomycetes, dinoflagellates, and ciliates, also possess more than one FVCC subfamily (Fig. 2), which indicates that not lesser signalling complexity can be expected for their cells. Precise data about the physiological role of FVCCs in protists can be obtained only by means of electrophysiological techniques, but their application to protists is not straightforward due to their specific cell coverings, motility, etc.^28,29^. However, analysis of the FVCC primary structure allows making predictions about their functioning through comparison with structural features of already characterised FVCCs.

Wide distribution of the selectivity filters E/E/E/E among FVCCs of distantly related eukaryotes indicates that it was likely a selectivity filter motif of the ancestral four-domain channel. Since E/E/E/E motif is typical for calcium-selective HVA Ca_v_ and calcium-permeable NALCN channels of metazoans, as well as for some characterised calcium channels of protists, we assume that the ancestral FVCC could be calcium-permeable or calcium-selective. Taking into account the uneven phylogenetic distribution of negatively charged amino acid residues in the positions n + 1 important to calcium selectivity, we propose that the ancestral FVCC was calcium-permeable rather than calcium-selective. However, it should be noted that, according to the recent study of the snail LVA Ca_v_ channel, selectivity of FVCCs may be determined not only by the structure of a selectivity filter and the nearest positions but also by the extracellular parts of P-loops^30^.

The acquiring of sodium selectivity by four-domain channels is one of the most intriguing gains in the course of FVCC evolution since it was presumably fundamental for differentiation of sodium and calcium signalling and further emergence of the nervous system in metazoans^31^. So far, four-domain sodium-selective channels have been identified only in this group of organisms. It was shown that sodium-selectivity/permeability of FVCCs emerged several times independently^14,15,30^. The presence of a lysine residue in a selectivity filter of FVCC is believed to determine sodium selectivity or at least permeability of such a channel^2^.

We identified lysine-containing selectivity filters in FVCC sequences of other eukaryotes. Several sequences of non-metazoan protists, i.e. Oomycota, Cryptophyta, and Apusomonadida, contain lysine in their selectivity filters (Fig. 3). Thus, they are candidates for sodium-selective/permeable channels, although experimental data about voltage-gated sodium channels in these organisms are absent. In addition, several unusual arginine- and histidine-containing selectivity filters have been identified in FVCCs of prasinophytes and the apusomonad *Thecamonas*. Similar to lysine, arginine is positively charged, and histidine can be positively charged at pH ≤ 7, but they structurally differ from the former. Currently, it is impossible to predict the selectivity type of arginine- and histidine-bearing selectivity filters because substitution of one amino acid residue of a filter with another may affect selectivity, even if structural differences between two residues are small.

Most FVCC sequences analysed in this study have all four arginine/lysine-rich S4s, suggesting that the ancestral FVCC also possessed four arginine/lysine-rich S4s and was probably voltage-sensitive. Nevertheless, some protistan lineages of channels have FVCCs with at least one arginine/lysine-poor segment S4 (Fig. 3, Supplementary Table S2). We assume that these channels are voltage-insensitive since characterised voltage-insensitive channels of metazoans (NALCN, Na_x_) and fungi (Cch) have arginine/lysine-poor S4s^11–13^. Apparently, the loss of positive charges in S4s took place several times in the course of FVCC evolution. Our results show that many unicellular eukaryotic organisms possess histidine-containing S4s. Interestingly, Na_x_ channels of mammals also possess this residue in D_IV_ S4^13^, but its role is unclear. It was shown that voltage-gated potassium channels bearing a histidine residue in S4s acquire an ability to conduct protons or other cations through their S1–S4 region^32,33^. The architecture of such channels is very similar to that of each FVCC domain^2^, which raises the question about possibly dual ion conductivity of protistan FVCCs with histidine-containing S4s provided by the conventional S5–P-loop–S6 region and by the S1–S4 region.

The canonical structure of Na_v_-like inactivation gates, i.e. the motif ΦΦΦT(S), is widely dispersed among various clades of FVCCs from evolutionary distant groups of organisms (Fig. 3, Supplementary Table S3), indicating that this motif emerged long before the appearance of Na_v_. Moreover, A and N residues of the inactivation gates docking site seem to be inherited from the ancestral FVCC (Supplementary Table S3). Thus, we assume that the ancestral FVCC already possessed ΦΦΦT(S) inactivation gates and A/X/N docking motifs. However, the canonical A/F/N motif representing an essential part of the inactivation gates docking site in Na_v_ subfamily seems to be a gain of metazoan Na_v_ channels (Supplementary Table S3).

It should be highlighted that the presence of inactivation gates in the channel structure does not guarantee fast inactivation. For instance, NALCN channels possess this motif but do not demonstrate the Na_v_-like type of fast inactivation. Other metazoan channels incapable of fast inactivation (Ca_v_1, LVA Ca_v_) or channels with a different mechanism of this process (e.g. some of Ca_v_2) bear a deletion of the entire inactivation gates motif or contain amino acid substitutions in its structure (polar or charged instead of hydrophobic residues in the ΦΦΦ triplet; a charged residue instead of T(S)). Similar substitutions appear in some channels of unicellular eukaryotes suggesting their inability for fast inactivation.

We screened FVCC sequences for the presence of other structures which may be involved in FVCC activity regulation, namely, AID typical to HVA Ca_v_ and pre-IQ and IQ motifs. We found out that only members of the HVA Ca_v_ clade bear AID. Hence, the appearance of AID in HVA Ca_v_ can be considered as a lineage-specific gain that led to their ability for interaction with Ca_v_β (Fig. 3). Motifs homologous to pre-IQ and IQ are widely spread among various clades of the FVCC phylogenetic tree indicating that the ancestral FVCC could potentially bind calmodulin.

In some cases, the information about phylogeny and functional determinants of protistan FVCCs can be compared to the existing electrophysiological data. Voltage-gated calcium channels from cilia of *Paramecium tetraurelia* with the selectivity filter E/E/E/E have been characterised experimentally^34^. These channels do not demonstrate Na_v_-like fast inactivation and bear calmodulin-binding sites. In the present work, we found FVCCs of *P. tetraurelia* from WSCs XXV and XXVI with putatively similar characteristics. At the same time, our analysis demonstrated that this organism may possess more types of FVCCs than have been shown by means of electrophysiology. For example, subclade Ic (Fig. 3) unites likely voltage-insensitive channels with the uncommon selectivity filter motif D/D/D/D. Moreover, some FVCCs from subclades Ia and Ic (Fig. 3) have other selectivity filters, e.g. E/S/L/L, E/Q/D/E, N/E/D/N, and N/D/D/N, all with an unknown selectivity type. Another trait of many FVCCs of *P. tetraurelia* is the loss of one of the calmodulin-binding sites, either pre-IQ (subclade Ic) or IQ (subclade Ia) motifs (Fig. 3).

Structural diversity is also typical for FVCCs of *Chlamydomonas reinhardtii*. The only characterised FVCC of this organism is a voltage-gated calcium-selective channel with the selectivity filter E/E/E/E^35^; however, our data demonstrate that *Ch. reinhardtii* has channels with both conventional E/E/E/E and unusual D/D/D/D and T/E/N/D selectivity filters. Significant variability of FVCCs found in *P. tetraurelia* and *Ch. reinhardtii* is likely to underpin complex physiological reactions of these organisms, e.g. various taxes and rhythmical beating of cilia and flagella.

## Conclusions

Apparently, FVCCs were present in the common ancestor of modern eukaryotes and rapidly evolved in the course of eukaryotic diversification. Our data on the primary structure of functional determinants of FVCCs overlain on their phylogeny indicate that the ancestral four-domain channel possessed: 1) selectivity filter characteristic to Ca_v_ (E/E/E/E); 2) four arginine/lysine-rich transmembrane segments S4; 3) inactivation gates characteristic to Na_v_; 4) pre-IQ and IQ motifs; and 5) no AID. For a long time, voltage-gated calcium channels have been considered ancestral to Na_v_ subfamily^9,31,36,37^. Our results suggest that a voltage-gated calcium-permeable channel was the common ancestor of all eukaryotic FVCCs, including five known genetic subfamilies (HVA Ca_v_, LVA Ca_v_, Na_v_, NALCN, Cch). This ancestral voltage-gated calcium channel already had a structural potential for the Na_v_-type fast inactivation and regulation by the ancient calmodulin mechanism, but could not interact with the auxiliary protein Ca_v_β. The evolution of this channel resulted in the present diversity of eukaryotic four-domain channels. Among the most significant events on this way were gains and losses of various functional determinants, such as appearance of a lysine residue in a selectivity filter, acquisition of AID, elimination of positively charged amino acids from S4, deletion of inactivation gates, and reduction of pre-IQ and/or IQ motifs, in various lineages of descendant FVCCs.

## Materials and Methods

### Databases Searches

The following databases were searched for FVCC sequences: GenBank, RefSeq, Origins of Multicellularity, UniProtKB/SwissProt, WormBase, as well as the database of Marine Microbial Eukaryote Transcriptome Sequencing Project (MMETSP; http://data.imicrobe.us/project/view/104, Combined Assemblies)^38^, i.e. transcriptomes Alexandrium-tamarense-CCMP1771, Amphidinium-carterae-CCMP1314, Chattonella-subsula-CCMP2191, Crypthecodinium-cohnii-Seligo, Gephyrocapsa-oceanica-RCC1303, Isochrysis-glabana-CCMP1323, Karenia-brevis-CCMP2229, Kryptoperidinium-foleaceum-CCMP1326, Lingulodinium-polyedra-CCMP1738, Lotharella-globoso-CCCM811, Oxyrrhis-marina-LB1974, Prorocentrum-minimum-CCMP1329, Prorocentrum-minimum-CCMP2233, Scrippsiella-trochoidea-CCMP3099, Symbiodinium-sp-Mp.

Since the goal of the present work was to investigate the phylogenetic diversity of eukaryotic FVCCs, we focused on protistan four-domain channels aiming to cover the maximal number of high-ranking eukaryotic groups. In some eukaryotic groups, such as dinoflagellates, ciliates, oomycetes and chlorophytes, we found more FVCC sequences than in others (e.g. haptophytes and cercozoans), probably due to the uneven representation of these groups in the available databases. In concordance with previous studies^9,10^, we did not reveal four-domain channels in embryophytes and amoebozoans. We intentionally used only a few representative sequences from each of the metazoan channel subfamilies (Na_v_, HVA Ca_v_, LVA Ca_v_, and NALCN). A deeper sampling of these subfamilies would lead to enlargement of the respective clades, but would not result in a better resolution of the entire tree, because it is known that metazoans possess only FVCCs from these four subfamilies. Moreover, phylogenetic relationships inside the metazoan Na_v_, HVA Ca_v_, LVA Ca_v_, and NALCN clades are well studied^3,6,8,10,20^.

Protein sequences of Na_v_, HVA Ca_v_, LVA Ca_v_, and NALCN of *Homo sapiens* and Cch of *Saccharomyces cerevisiae* were used as queries in BLASTp^39^ searches (BLOSUM62, E-value ≤ 10^−25^) against listed databases. In order to identify more homologues in protists, hits from the first BALSTp output were used as queries in the repeated BLASTp searches. To identify FVCC homologues in dinoflagellates, we used previously revealed FVCC sequences of the species *Prorocentrum minimum* as BLASTp queries^40^.

### Multiple Sequence Alignment and Phylogeny Reconstruction

Protein sequences containing four conservative domains pfam00520 (as referred in the database of conservative protein domains Pfam^41^ were selected for phylogenetic analysis. The domain pfam00520 corresponds to S1–S6 segments of FVCCs. Sequences bearing incomplete first pfam-domain (only S5–P-loop–S6) were also chosen. The final dataset consisted of 277 FVCC amino acid sequences from different groups of eukaryotes (Supplementary Table S1) which were then aligned using MAFFT7^42^ (BLOSUM62, FFT-NS-I). Conservative domains and functionally important regions of uncharacterised channels were predicted using the MAFFT alignment.

The obtained multiple alignment was improved manually and automatically trimmed using Gblocks software^43^. The best-fit evolution model was chosen by means of ProtTest^44^ according to the minimum -lnL value: LG + Г_4_ + F. The final alignment was used to reconstruct the phylogeny of FVCCs. Maximal likelihood and Bayesian analyses were conducted in Garli 2.1^45^ and MrBayes 3.2.5^46^ (four Markov chains, 5000000 generations with the tree sampling frequency of 5000 and burning fraction 25%), respectively. Bootstrapping was performed using 1000 pseudo-replicates. Bootstrap values ≥ 70 are usually considered as a minimal threshold of confidence^47^. Due to the high degree of FVCC variability, in the present work bootstrap values ≥ 65 and posterior probabilities ≥ 0.95 were considered as confident. The obtained phylogenetic trees were visualised in FigTree 1.4.2^48^. An additional maximal likelihood analysis (IQ-tree^49^) was performed using the same evolution model but an alternative alignment (MUSCLE^50^) automatically trimmed by means of Gblocks software^43^ (Supplementary Fig. S1). This extra validation step confirmed our results: overall, the tree topology remained unchanged. It should be noted, that in some cases this analysis resulted in somewhat higher bootstrap values.

### Data availability

The datasets generated and analysed during this study are available from the corresponding author upon reasonable request.

## Electronic supplementary material

Supplementary Information
