# Supplementary material for: Diversity and evolution of four-domain voltage-gated cation channels of eukaryotes and their ancestral functional determinants
Source: Sci Rep. 2018 Feb 23;8:3539. doi: 10.1038/s41598-018-21897-7 (PMC5824947; doi:10.1038/s41598-018-21897-7)
Supplement: Supplementary file 1 — Supplementary Information [file 41598_2018_21897_MOESM1_ESM.pdf]

Supplementary Information

Diversity and evolution of four-domain voltage-gated cation channels of eukaryotes and their ancestral functional determinants

Ilya Pozdnyakov*, Olga Matantseva, Sergei Skarlato

Institute of Cytology, Russian Academy of Sciences, Saint Petersburg, 194064, Russia

***Corresponding author**: E-mail: pozdnyakov@incras.ru

№	Seq. name	Organism	Accession number	Data base
**SAR**				
ALVEOLATA				
Dinoflagellata				
1	At406917	*Alexandrium tamarense* CCMP1771	406917_1	MMETSP
2	Ac156993	*Amphidinium carterae* CCMP1314	156993_1	MMETSP
3	Ac29873	*Amphidinium carterae* CCMP1314	29873_1	MMETSP
4	Kb20887	*Karinia brevis* CCMP2229	20887_1	MMETSP
5	Kb269344	*Karinia brevis* CCMP2229	269344_1	MMETSP
6	Kb53452	*Karinia brevis* CCMP2229	53452_1	MMETSP
7	Kb269973	*Karinia brevis* CCMP2229	269973_1	MMETSP
8	Kb20579	*Karinia brevis* CCMP2229	20579_1	MMETSP
9	Kf405392	*Kryptoperidinium foleaceum* CCMP1326	405392_1	MMETSP
10	Kf407651	*Kryptoperidinium foleaceum* CCMP1326	407651_1	MMETSP
11	Kf416989	*Kryptoperidinium foleaceum* CCMP1326	416989_1	MMETSP
12	Lp90575	*Lingulodinium polyedra* CCMP1738	90575_1	MMETSP
13	Om66755	*Oxyrrhis marina* LB1974	66755_1	MMETSP
14	Pm40145	*Prorocentrum minimum* CCMP2233	40145_1	MMETSP
15	Pm47759	*Prorocentrum minimum* CCMP2233	47759_1	MMETSP
16	Pm20998	*Prorocentrum minimum* CCMP2233	20998_1	MMETSP
17	Pm2595	*Prorocentrum minimum* CCMP2233	2595_1	MMETSP
18	St17126	*Scrippsiella trochoidea* CCMP3099	17126_1	MMETSP
19	St9808	*Scrippsiella trochoidea* CCMP3099	9808_1	MMETSP
20	St26908	*Scrippsiella trochoidea* CCMP3099	26908_1	MMETSP
21	St388392	*Scrippsiella trochoidea* CCMP3099	388392_1	MMETSP
22	St391309	*Scrippsiella trochoidea* CCMP3099	391309_1	MMETSP
23	Ssp188361	*Symbiodinium sp* Mp	188361_1	MMETSP
24	Ssp190870	*Symbiodinium sp* Mp	190870_1	MMETSP
Perkinsozoa				
25	Perkim1	*Perkinsus marinus*	XP_002776392.1	NCBI RefSeq

№	Seq. name	Organism	Accession number	Data base
26	Perkim2	*Perkinsus marinus*	XP_002788713.1	NCBI RefSeq
		Apicomplexa		
27	Toxopg1	*Toxoplasma gondii*	EPT29988.1	GenBank
28	Toxopg2	*Toxoplasma gondii*	ESS33237.1	GenBank
		Chromerida		
29	Vitreb1	*Vitrella brassicaformis*	CEM06448.1	GenBank
30	Vitreb2	*Vitrella brassicaformis*	CEM24784.1	GenBank
31	Vitreb3	*Vitrella brassicaformis*	CEM21952.1	GenBank
32	Vitreb4	*Vitrella brassicaformis*	CEM11618.1	GenBank
		Ciliata		
33	Ichthm1	*Ichthyophthirius multifiliis*	XP_004035037.1	NCBI RefSeq
34	Ichthm2	*Ichthyophthirius multifiliis*	XP_004037277.1	NCBI RefSeq
35	Ichthm3	*Ichthyophthirius multifiliis*	XP_004035093.1	NCBI RefSeq
36	Ichthm4	*Ichthyophthirius multifiliis*	XP_004036524.1	NCBI RefSeq
37	Ichthm5	*Ichthyophthirius multifiliis*	XP_004024005.1	NCBI RefSeq
38	Oxytrt1	*Oxytricha trifallax*	EJY83225.1	GenBank
39	Oxytrt2	*Oxytricha trifallax*	EJY65938.1	GenBank
40	Oxytrt3	*Oxytricha trifallax*	EJY81393.1	GenBank
41	Oxytrt4	*Oxytricha trifallax*	EJY83098.1	GenBank
42	Oxytrt5	*Oxytricha trifallax*	EJY85379.1	GenBank
43	Oxytrt6	*Oxytricha trifallax*	EJY78158.1	GenBank
44	Oxytrt7	*Oxytricha trifallax*	EJY69333.1	GenBank
45	Oxytrt8	*Oxytricha trifallax*	EJY78389.1	GenBank
46	Oxytrt9	*Oxytricha trifallax*	EJY84542.1	GenBank
47	Oxytrt10	*Oxytricha trifallax*	EJY64416.1	GenBank
48	Oxytrt11	*Oxytricha trifallax*	EJY78675.1	GenBank
49	Oxytrt12	*Oxytricha trifallax*	EJY66049.1	GenBank
50	Oxytrt13	*Oxytricha trifallax*	EJY79258.1	GenBank
51	Paramt1	*Paramecium tetraurelia*	XP_001445891.1	NCBI RefSeq
52	Paramt2	*Paramecium tetraurelia*	XP_001458988.1	NCBI RefSeq
53	Paramt3	*Paramecium tetraurelia*	XP_001429788.1	NCBI RefSeq
54	Paramt4	*Paramecium tetraurelia*	XP_001432429.1	NCBI RefSeq
55	Paramt5	*Paramecium tetraurelia*	XP_001450373.1	NCBI RefSeq
56	Paramt6	*Paramecium tetraurelia*	XP_001441486.1	NCBI RefSeq
57	Paramt7	*Paramecium tetraurelia*	XP_001441606.1	NCBI RefSeq
58	Paramt8	*Paramecium tetraurelia*	XP_001453058.1	NCBI RefSeq
59	Paramt9	*Paramecium tetraurelia*	XP_001450374.1	NCBI RefSeq
60	Paramt10	*Paramecium tetraurelia*	XP_001452932.1	NCBI RefSeq
61	Paramt11	*Paramecium tetraurelia*	XP_001424527.1	NCBI RefSeq
62	Paramt12	*Paramecium tetraurelia*	XP_001457036.1	NCBI RefSeq
63	Paramt13	*Paramecium tetraurelia*	XP_001424021.1	NCBI RefSeq
64	Paramt14	*Paramecium tetraurelia*	XP_001455088.1	NCBI RefSeq
65	Paramt15	*Paramecium tetraurelia*	XP_001435951.1	NCBI RefSeq
66	Paramt16	*Paramecium tetraurelia*	XP_001455025.1	NCBI RefSeq
67	Paramt17	*Paramecium tetraurelia*	XP_001430688.1	NCBI RefSeq

№	Seq. name	Organism	Accession number	Data base
68	Paramt18	*Paramecium tetraurelia*	XP_001435671.1	NCBI RefSeq
69	Paramt19	*Paramecium tetraurelia*	XP_001445828.1	NCBI RefSeq
70	Paramt20	*Paramecium tetraurelia*	XP_001450103.1	NCBI RefSeq
71	Paramt21	*Paramecium tetraurelia*	XP_001455210.1	NCBI RefSeq
72	Paramt22	*Paramecium tetraurelia*	XP_001445200.1	NCBI RefSeq
73	Paramt23	*Paramecium tetraurelia*	XP_001424033.1	NCBI RefSeq
74	Paramt24	*Paramecium tetraurelia*	XP_001430317.1	NCBI RefSeq
75	Paramt25	*Paramecium tetraurelia*	XP_001455219.1	NCBI RefSeq
76	Paramt26	*Paramecium tetraurelia*	XP_001433501.1	NCBI RefSeq
77	Paramt27	*Paramecium tetraurelia*	XP_001435405.1	NCBI RefSeq
78	Paramt28	*Paramecium tetraurelia*	XP_001438028.1	NCBI RefSeq
79	Paramt29	*Paramecium tetraurelia*	XP_001430212.1	NCBI RefSeq
80	Paramt30	*Paramecium tetraurelia*	XP_001445201.1	NCBI RefSeq
81	Paramt31	*Paramecium tetraurelia*	XP_001450132.1	NCBI RefSeq
82	Paramt32	*Paramecium tetraurelia*	XP_001424019.1	NCBI RefSeq
83	Paramt33	*Paramecium tetraurelia*	XP_001430323.1	NCBI RefSeq
84	Paramt34	*Paramecium tetraurelia*	XP_001438029.1	NCBI RefSeq
85	Stylol1	*Stylonychia lemnae*	CDW88224.1	GenBank
86	Stylol2	*Stylonychia lemnae*	CDW78331.1	GenBank
87	Stylol3	*Stylonychia lemnae*	CDW78622.1	GenBank
88	Stylol4	*Stylonychia lemnae*	CDW83245.1	GenBank
89	Stylol5	*Stylonychia lemnae*	CDW86413.1	GenBank
90	Stylol6	*Stylonychia lemnae*	CDW82972.1	GenBank
91	Stylol7	*Stylonychia lemnae*	CDW81684.1	GenBank
92	Stylol8	*Stylonychia lemnae*	CDW88937.1	GenBank
93	Stylol10	*Stylonychia lemnae*	CDW87516.1	GenBank
94	Stylol111	*Stylonychia lemnae*	CDW78731.1	GenBank
95	Tetrat1	*Tetrahymena thermophila*	XP_001022128.2	NCBI RefSeq
96	Tetrat2	*Tetrahymena thermophila*	XP_012654668.1	NCBI RefSeq
97	Tetrat3	*Tetrahymena thermophila*	XP_001015043.2	NCBI RefSeq
98	Tetrat4	*Tetrahymena thermophila*	XP_001022123.2	NCBI RefSeq
99	Tetrat6	*Tetrahymena thermophila*	XP_001010839.2	NCBI RefSeq
100	Tetrat7	*Tetrahymena thermophila*	XP_976857.2	NCBI RefSeq
101	Tetrat8	*Tetrahymena thermophila*	XP_976859.2	NCBI RefSeq
102	Tetrat9	*Tetrahymena thermophila*	XP_001010833.2	NCBI RefSeq
STRAMENOPILES				
Bacillariophyta				
103	Thalap1	*Thalassiosira pseudonana*	XP_002289136.1	NCBI RefSeq
Phaeophyta				
104	Ectocs1	*Ectocarpus siliculosus*	CBJ26318.1	GenBank
Raphidophyta				
105	Chatts1	*Chattonella subsula* CCMP2191	3660_1	MMETSP
106	Chatts2	*Chattonella subsula* CCMP2191	15938_1	MMETSP
107	Chatts3	*Chattonella subsula* CCMP2191	21045_1	MMETSP
108	Chatts4	*Chattonella subsula* CCMP2191	208558_1	MMETSP

№	Seq. name	Organism	Accession number	Data base
109	Chatts5	*Chattonella subsula* CCMP2191	208266_1	MMETSP
110	Chatts6	*Chattonella subsula* CCMP2191	208985_1	MMETSP
		Oomycota		
111	Albugc1	*Albugo candida*	CCI44742.1	GenBank
112	Albugc2	*Albugo candida*	CCI44287.1	GenBank
113	Albugc3	*Albugo candida*	CCI44953.1	GenBank
114	Albugl1	*Albugo laibachii*	CCA18882.1	GenBank
115	Albugl2	*Albugo laibachii*	CCA23261.1	GenBank
116	Albugl3	*Albugo laibachii*	CCA21894.1	GenBank
117	Aphana1	*Aphanomyces astaci*	XP_009839617.1	NCBI RefSeq
118	Aphana2	*Aphanomyces astaci*	XP_009832906.1	NCBI RefSeq
119	Aphana3	*Aphanomyces astaci*	XP_009839619.1	NCBI RefSeq
120	Aphana4	*Aphanomyces astaci*	XP_009832839.1	NCBI RefSeq
121	Aphana5	*Aphanomyces astaci*	XP_009845434.1	NCBI RefSeq
122	Aphana6	*Aphanomyces astaci*	XP_009823311.1	NCBI RefSeq
123	Aphana7	*Aphanomyces astaci*	XP_009839618.1	NCBI RefSeq
124	Aphana8	*Aphanomyces astaci*	XP_009845430.1	NCBI RefSeq
125	Aphana9	*Aphanomyces astaci*	XP_009839621.1	NCBI RefSeq
126	Aphani1	*Aphanomyces invadans*	XP_008874382.1	NCBI RefSeq
127	Aphani2	*Aphanomyces invadans*	XP_008874434.1	NCBI RefSeq
128	Aphani4	*Aphanomyces invadans*	XP_008866219.1	NCBI RefSeq
129	Aphani5	*Aphanomyces invadans*	XP_008861253.1	NCBI RefSeq
130	Aphani6	*Aphanomyces invadans*	XP_008861246.1	NCBI RefSeq
131	Phytoi1	*Phytophthora infestans*	XP_002902845.1	NCBI RefSeq
132	Phytoi2	*Phytophthora infestans*	XP_002906745.1	NCBI RefSeq
133	Phytoi3	*Phytophthora infestans*	XP_002897879.1	NCBI RefSeq
134	Phytop1	*Phytophthora parasitica*	ETM31623.1	GenBank
135	Phytop2	*Phytophthora parasitica*	ETI46994.1	GenBank
136	Phytop3	*Phytophthora parasitica*	XP_008907518.1	NCBI RefSeq
137	Phytop4	*Phytophthora parasitica*	XP_008902841.1	NCBI RefSeq
138	Phytop5	*Phytophthora parasitica*	ETO61255.1	GenBank
139	Phytos1	*Phytophthora sojae*	XP_009533666.1	NCBI RefSeq
140	Phytos2	*Phytophthora sojae*	XP_009533629.1	NCBI RefSeq
141	Phytos3	*Phytophthora sojae*	XP_009533651.1	NCBI RefSeq
142	Phytos4	*Phytophthora sojae*	XP_009515830.1	NCBI RefSeq
143	Phytos5	*Phytophthora sojae*	XP_009520661.1	NCBI RefSeq
144	Phytos6	*Phytophthora sojae*	XP_009515784.1	NCBI RefSeq
145	Phytos7	*Phytophthora sojae*	XP_009533630.1	NCBI RefSeq
146	Phytos8	*Phytophthora sojae*	XP_009523036.1	NCBI RefSeq
147	Saprod1	*Saprolegnia diclina*	XP_008617256.1	NCBI RefSeq
148	Saprod2	*Saprolegnia diclina*	XP_008613137.1	NCBI RefSeq
149	Saprod3	*Saprolegnia diclina*	XP_008607900.1	NCBI RefSeq
150	Saprod4	*Saprolegnia diclina*	XP_008603782.1	NCBI RefSeq
151	Saprod5	*Saprolegnia diclina*	XP_008615211.1	NCBI RefSeq
152	Saprod6	*Saprolegnia diclina*	XP_008610030.1	NCBI RefSeq
153	Saprod7	*Saprolegnia diclina*	XP_008615216.1	NCBI RefSeq
154	Saprop1	*Saprolegnia parasitica*	XP_012205304.1	NCBI RefSeq

№	Seq. name	Organism	Accession number	Data base
155	Saprop2	*Saprolegnia parasitica*	XP_012198411.1	NCBI RefSeq
156	Saprop4	*Saprolegnia parasitica*	XP_012196164.1	NCBI RefSeq
157	Saprop5	*Saprolegnia parasitica*	XP_012199523.1	NCBI RefSeq
158	Saprop6	*Saprolegnia parasitica*	XP_012194876.1	NCBI RefSeq
159	Saprop7	*Saprolegnia parasitica*	XP_012196160.1	NCBI RefSeq
RHIZARIA				
Cercozoa				
160	Lothag1	*Lotharella globoso* CCCM811	120725_1	MMETSP
161	Lothag2	*Lotharella globoso* CCCM811	121633_1	MMETSP
162	Plasmb1	*Plasmodiophora brassicae*	CEP00345.1	GenBank
‘HACROBIA’				
Haptophyta				
163	Chryss1	*Chrysochromulina sp.*	KOO23505.1	GenBank
164	Chryss2	*Chrysochromulina sp.*	KOO25755.1	GenBank
165	Gephyo1	*Gephyrocapsa oceanica* RCC1303	2028_1	MMETSP
166	Gephyo2	*Gephyrocapsa oceanica* RCC1303	151358_1	MMETSP
167	Isochg1	*Isochrysis galbana* CCMP1323	1047_1	MMETSP
168	Isochg2	*Isochrysis galbana* CCMP1323	26711_1	MMETSP
169	Isochg3	*Isochrysis galbana* CCMP1323	37836_1	MMETSP
170	Isochg4	*Isochrysis galbana* CCMP1323	107351_1	MMETSP
Cryptophyta				
171	Guillt1	*Guillardia theta*	XP_005827187.1	NCBI RefSeq
172	Guillt2	*Guillardia theta*	XP_005831410.1	NCBI RefSeq
173	Guillt3	*Guillardia theta*	XP_005833047.1	NCBI RefSeq
174	Guillt4	*Guillardia theta*	XP_005834581.1	NCBI RefSeq
175	Guillt5	*Guillardia theta*	XP_005828971.1	NCBI RefSeq
176	Guillt6	*Guillardia theta*	XP_005824876.1	NCBI RefSeq
177	Guillt7	*Guillardia theta*	XP_005840919.1	NCBI RefSeq
178	Guillt8	*Guillardia theta*	XP_005829415.1	NCBI RefSeq
179	Guillt9	*Guillardia theta*	XP_005822036.1	NCBI RefSeq
180	Guillt10	*Guillardia theta*	XP_005827163.1	NCBI RefSeq
ARHAEPLASTIDA				
Chlorophyta				
181	Bathyp1	*Bathycoccus prasinos*	XP_007508934.1	NCBI RefSeq
182	Bathyp2	*Bathycoccus prasinos*	XP_007515549.1	NCBI RefSeq
183	Chlamr1	*Chlamydomonas reinhardtii*	XP_001701446.1	NCBI RefSeq
184	Chlamr2	*Chlamydomonas reinhardtii*	BAH15384.1	GenBank
185	Chlamr3	*Chlamydomonas reinhardtii*	XP_001690493.1	NCBI RefSeq

№	Seq. name	Organism	Accession number	Data base
186	Chlamr4	*Chlamydomonas reinhardtii*	XP_001691131.1	NCBI RefSeq
187	Chlamr5	*Chlamydomonas reinhardtii*	XP_001692115.1	NCBI RefSeq
188	Chlamr6	*Chlamydomonas reinhardtii*	XP_001692912.1	NCBI RefSeq
189	Chlorv1	*Chlorella variabilis*	XP_005847467.1	NCBI RefSeq
190	Microp1	*Micromonas pusilla*	XP_003062267.1	NCBI RefSeq
191	Microp2	*Micromonas pusilla*	XP_003056815.1	NCBI RefSeq
192	Microp3	*Micromonas pusilla*	XP_003060664.1	NCBI RefSeq
193	Microp4	*Micromonas pusilla*	XP_003062007.1	NCBI RefSeq
194	Microp5	*Micromonas pusilla*	XP_003058693.1	NCBI RefSeq
195	Micros1	*Micromonas sp.* RCC229	XP_002508803.1	NCBI RefSeq
196	Micros2	*Micromonas sp.* RCC229	XP_002502721.1	NCBI RefSeq
197	Micros3	*Micromonas sp.* RCC229	XP_002507789.1	NCBI RefSeq
198	Micros4	*Micromonas sp.* RCC229	XP_002503057.1	NCBI RefSeq
199	Micros5	*Micromonas sp.* RCC229	XP_002504429.1	NCBI RefSeq
200	Micros6	*Micromonas sp.* RCC229	XP_002504950.1	NCBI RefSeq
201	Micros7	*Micromonas sp.* RCC229	XP_002508124.1	NCBI RefSeq
202	Micros8	*Micromonas sp.* RCC229	XP_002508756.1	NCBI RefSeq
203	Monorn1	*Monoraphidium neglectum*	XP_013905746.1	NCBI RefSeq
204	Ostrel1	*Ostreococcus lucimarinus*	XP_001418450.1	NCBI RefSeq
205	Ostrel2	*Ostreococcus lucimarinus*	XP_001415426.1	NCBI RefSeq
206	Ostrel3	*Ostreococcus lucimarinus*	XP_001422214.1	NCBI RefSeq
207	Ostret1	*Ostreococcus tauri*	CEF98340.1	GenBank
208	Ostret2	*Ostreococcus tauri*	CEF97603.1	GenBank
209	Ostret3	*Ostreococcus tauri*	CEF96560.1	GenBank
210	Ostret4	*Ostreococcus tauri*	XP_003074718.1	NCBI RefSeq
211	Ostret5	*Ostreococcus tauri*	XP_003078817.1	NCBI RefSeq
212	Volvoc1	*Volvox carteri*	XP_002958358.1	NCBI RefSeq
213	Volvoc2	*Volvox carteri*	XP_002959193.1	NCBI RefSeq
214	Volvoc3	*Volvox carteri*	XP_002954479.1	NCBI RefSeq
215	Volvoc4	*Volvox carteri*	XP_002950273.1	NCBI RefSeq
EXCAVATA				
Euglenozoa, Kinetoplastida				
216	Leishb1	*Leishmania braziliensis*	XP_001564333.2	NCBI RefSeq
217	Leishi1	*Leishmania infantum*	XP_001468437.1	NCBI RefSeq
218	Leishm1	*Leishmania mexicana*	XP_003878633.1	NCBI RefSeq
219	Leptos1	*Leptomonas seymouri*	KPI83192.1	GenBank
220	Leptos2	*Leptomonas seymouri*	KPI89096.1	GenBank
221	Phytosp1	*Phytomonas sp.*	CCW67620.1	GenBank
222	Trypab1	*Trypanosoma brycei*	XP_822541.1	NCBI RefSeq
223	Trypac1	*Trypanosoma cruzi*	EKF32800.1	GenBank
224	Trypac2	*Trypanosoma cruzi*	XP_819699.1	NCBI RefSeq
225	Trypag1	*Trypanosoma grayi*	XP_009306110.1	NCBI RefSeq
226	Trypag2	*Trypanosoma grayi*	XP_009308995.1	NCBI RefSeq
227	Trypar1	*Trypanosoma rangeli*	ESL05805.1	GenBank
228	Trypav1	*Trypanosoma vivax*	CCC51237.1	GenBank

№	Seq. name	Organism	Accession number	Data base
**OBAZOA**				
APUSOZOA				
Apusomonadida				
229	Thecat1	*Thecamonas trahens*	XP_013760129.1	NCBI RefSeq
230	Thecat2	*Thecamonas trahens*	AMSG_03029.2	Origins of Multicellularity
231	Thecat3	*Thecamonas trahens*	AMSG_03189.2	Origins of Multicellularity
232	Thecat4	*Thecamonas trahens*	AMSG_05523.2	Origins of Multicellularity
233	Thecat5	*Thecamonas trahens*	AMSG_06300.2	Origins of Multicellularity
234	Thecat6	*Thecamonas trahens*	AMSG_06944.2	Origins of Multicellularity
235	Thecat7	*Thecamonas trahens*	AMSG_03287.2	Origins of Multicellularity
OPISTHOKONTA				
Metazoa (Na_v1)				
236	AplyNav1	*Aplysia californica*	AAC47457.1	GenBank
237	BranNav1	*Branchiostoma floridae*	AEX00069.1	GenBank
238	DrosNav1	*Drosophila melanogaster*	AAB59195.1	GenBank
239	HaloNav1	*Halocynthia roretzi*	BAA04133.1	GenBank
240	HomoNav1	*Homo sapiens*	NP_001189364.1	NCBI RefSeq
241	MusmNav1	*Mus musculus*	NM_133199.2	NCBI RefSeq
Metazoa (Na_v2)				
242	BranNav2	*Branchiostoma floridae*	AEX00068.1	GenBank
243	CyanNav	*Cyanea capillata*	AAA75572.1	GenBank
244	DrosNav2	*Drosophila melanogaster*	Q9W0Y8.5	UniProtKB/Swiss-Prot
245	ExaiNav	*Exaiptasia pallida*	AAB96953.1	GenBank
246	HaloNav2	*Halocynthia roretzi*	BAA95896.1	GenBank
247	MnemNav	*Mnemiopsis leidyi alfa*	AEF59085.1	GenBank
248	NemaNav2	*Nematostella vectensis*	AEX00070.1	GenBank
249	PolyNav	*Polyorchis penicillatus*	AAC38974.1	GenBank
250	StroNav2	*Strongylocentrotus purpuratus*	XP_001189610.1	NCBI RefSeq
251	TricNav2	*Trichoplax adhaerens*	AEX00065.1	GenBank
Choanoflagellata (Na_v)				
252	MonoNav	*Monosiga brevicollis*	AEF59076.1	GenBank
Metazoa (HVA Ca_v: Ca_v1)				
253	CyanCavL	*Cyanea capillata*	AAC63050.1	GenBank
254	DrosCavL	*Drosophila melanogaster*	AAA81883.1	GenBank
255	HaloCavL	*Halocynthia roretzi*	BAA34927.2	GenBank
256	HomoCavL	*Homo sapiens*	Q13936.4	UniProtKB/Swiss-Prot
257	StylCavL	*Stylophora pistillata*	AAD11470.1	GenBank
Metazoa (HVA Ca_v: Ca_v2)				
258	ApisCav2	*Apis mellifera*	XP_006558026.1	NCBI RefSeq
259	CaenCav2	*Caenorhabditis elegans*	WP:CE31225	WormBase
260	HomoCavN	*Homo sapiens*	NP_000709.1	NCBI RefSeq

№	Seq. name	Organism	Accession number	Data base
261	HomCavPQ	*Homo sapiens*	NP_001167551.1	NCBI RefSeq
262	HomoCavR	*Homo sapiens*	NP_001192222.1	NCBI RefSeq
263	SchiCav2	*Schistosoma mansoni*	AAK84313.1	GenBank
Choanoflagellata (HVA Ca_v)				
264	SalpCavL	*Salpingoeca rosetta*	PTSG_09464.1	Origins of Multicellularity
Metazoa (LVA Ca_v)				
265	CaenCavT	*Caenorhabditis elegans*	WP:CE36117	WormBase
266	DrosCavT	*Drosophila melanogaster*	NP_001096889.1	NCBI RefSeq
267	HomoCavT	*Homo sapiens*	O95180.4	UniProtKB/Swiss-Prot
268	LymnCavT	*Lymnaea stagnalis*	AAO83843.2	GenBank
Metazoa (NALCN)				
269	CaeNALCN	*Caenorhabditis elegans*	NP_741413.2	NCBI RefSeq
270	DroNALCN	*Drosophila melanogaster*	NP_727772.2	NCBI RefSeq
271	HomNALCN	*Homo sapiens*	NP_443099.1	NCBI RefSeq
272	NemNALCN	*Nematostella vectensis*	XP_001637238.1	NCBI RefSeq
Fungi (Cch)				
273	AspeCch	*Aspergillus niger*	EHA17962.1	GenBank
274	PeniCch	*Penicillium brasilianum*	CEO58800.1	GenBank
275	PseuCch	*Pseudozyma antarctica*	XP_014654795.1	NCBI RefSeq
276	SaccCch	*Saccharomyces cerevisiae*	NP_011733.3	NCBI RefSeq
277	TrichCch	*Trichosporon oleaginosus*	KLT42032.1	GenBank

Table S1. List of amino acid sequences of four-domain voltage-gated cation channels used in the analyses.

№	Name	S4-D$_I$	S4-D$_{II}$	S4-D$_{III}$	S4-D$_{IV}$
**SAR**					
ALVEOLATA					
Dinoflagellata					
1	At406917	*Not analysed*	SALRTFRLFRVFKLAR	RSLRTLRALRPLRVISR	IVRTFRIGRIFRLVKS
2	Ac156993	GFLRMFRMLRPLRSLNA	SVLRVARLMRILNKIAS	KTLRILRALRPLRVISR	LLRVLRIFRLFRLLRF
3	Ac29873	*Not analysed*	SSLRAFRLLRIFKLAK	QIFRLARVLRPLRLISR	AFRIFRIARLFRLLRF
4	Kb20887	NILRVFRVLRPLRSINA	RAFRGFRLVRVVFKIAR	KNLRILRAFRPLRAISR	VIRIFRIARLFRLIRF
5	Kb269344	SSLRIFRALRPLRSMTM	TVLRIFRLARVVVKISN	KTLRILRAFRPLRVIAR	VIRIFRVARLFRLLRF
6	Kb53452	RFLLVFKVLRPLRSLTV	RAFRGLRLLRIFKLAK	KTLRIIRALRPLRVVAR	AIRAFRIARLFRLVRF
7	Kb269973	GSLRTVRILRPLRTINR	TVLRTFRLMRVFKLAR	KALRAIRALRPLRMVSR	MLRILRAARMFRLVKS
8	Kb20579	LLVRSVRILRPLRTINK	SALRVSRLLRIFKIFG	RAMRLLRVMRPLRLVRR	VLHAFRVMRVIRLVRV
9	Kf405392	GFLRLFRVLRPLRSLNA	KALRTLRLFRVLNKFAS	KTLRILRAFRPLRVIQR	VIRIFRIARLFRLLKF
10	Kf407651	GFLRLFRVLRPLRSLNS	QALRTLRLFRVLNKFAS	KTLRLLRAFRPLRVVKR	VVRIFRIARLFRLLRF
11	Kf416989	SFLRLFRVLRPLRSLNA	KAMRTLRLFRVLNKLAT	KTLRILRAFRPLRVINR	VLRIFRIARLFRLLKY
12	Lp90575	NFLRLFRLLRPLRSLNA	SALRTFRLFRVMNKLAN	RVLRILRTFRPLRVISR	VIRILRIARLFRLLRF
13	Om66755	SSLRSFRVLRPLRSLTV	SALRGLRLLRVFKLAK	KTIRILRALRPLRLINR	VIGVLRTLRLFRLVRF
14	Pm40145	GLPRTCHRAARPRPQERQRDQ	TALRTLRLFRVLNKLAS	KTLRILRALRPLRVISR	VVRIFRIARLFRLLRF
15	Pm47759	EYWRV------------	SVLRTFRLFRVMNKLAH	KTLRILRTFRPLRVVSR	ALQVL-----------
16	Pm20998	*Not analysed*	TALRVLRLFRVLNKLAY	KTLRILRAFRPLRVIAR	VIRIFRIARLFRLLRF
17	Pm2595	SFLRVFKVLRPLRSLNK	TALRVLRLFRVLNKLAY	KTLRILRAFRPLRVIAR	VIRIFRIARLFRLLRF
18	St17126	ILVRALRVLRPLRTINA	SALRIARLLRVFKIIG	RALRVVRVLRPLKLIKT	ILRGFRVLRVLRMARY
19	St9808	GFLRLFRVLRPLRSLNA	QALRTLRLFRVLNKFAS	KTLRILRAFRPLRVVKR	VIRIFRIARLFRLLKF
20	St26908	GFLRLFRVLRPLRSLNA	KALRTLRLFRVLNKFAS	KTLRILRAFRPLRVIKR	VIRIFRIARLFRLLKF
21	St388392	GFLRLFRILRPLRSLNA	TALRTLRLFRVLNKLAN	KTLRILRAFRPLRVISR	IIRIFRIGRLFR---F
22	St391309	SFIRTIRVLRPLRTLSA	TALRAFRLLRIFKLAK	KIFRIARMLRPLRIISR	AIRIFRVARLFRLVRF
23	Ssp188361	GFLRMFRILRPLRSLNA	TALRTLRLFRVLNKLAS	KTVRMLRALRPLRIISR	VTSLFRVARLFRLLHY
24	Ssp190870	GFFRVFRALRPLRSLNA	TAFRTLRLFRVLNKLAN	KTLRILRAFRPLRVISR	VIRIFRIARLFRLLRF
Perkinsozoa					
25	Perkim1	SVLRVFRVLRPLRSLTV	TALRGFRLLRIFKLAK	KTLRMLRALRPLRVISR	AIRIFRIARLFRLVRF
26	Perkim2	SVLRVFRVLRPLRSLTV	TALRGFRLLRIFKLAK	-----------LRVISR	AIRIFRIARLFRLVRF
Apicomplexa					
27	Toxopg1	VALFFELVRKLRVLRLFETKTCKL	DVVKTVDLVLNVLHSFRLFKLVR	RVVKVLRSLRSFLLFSL	LWRL.9.LSFRLFRVARFLYR
28	Toxopg2	VALFFELVRKLRVLRLFETKTCKL	DVVKTVDLVLNVLHSFRLFKLVR	RVVKVLRSLRSFLLFSL	LWRL.9.LSFRLFRVARFLYR

№	Name	S4-D_I	S4-D_{II}	S4-D_{III}	S4-D_{IV}
			Chromerida		
29	Vitreb1	SALRTIRLLRPLRTINR	TALRTFRLLRVLRLAR	KSLRAMRALRPLRMVSR	ILRILRIARLFRLIKS
30	Vitreb2	NVLRTFRAFRPLRSLNA	SAFRTFRLLRILKLAR	RALRVLKSFRAFRPLRIISR	MLRAFRIGRVLRLIRQ
31	Vitreb3	----IFRDLRPLRILSA	TALRAFRLFRIFRLAK	KSLRAFRALRPLRLISR	VVRVFRIARLFRLIRF
32	Vitreb4	TVIRTFR----------	GALRAFRLLRVLKLGR	RTLRAFRALRPLRIISR	M-------RSARLLQR
			Ciliata		
33	Ichthm1	NALRVLRILKSLKNINA	TSLRSLRLFRIFKFAR	QVFRLLRVLRPFRLISK	FIIIFRIVRVLRLIKK
34	Ichthm2	SALRSLRVLRPLRTISS	SAFRSVRILRVFRVLRVTRLIR	RILRLLRTLRPLRFISH	VLRVLRVTRLFRIIKT
35	Ichthm3	SSLRSLRILRPLRTIST	TAFKSIRILRVFRVLRVARLVR	KILRLLRTLRPLRFISH	VFRVLKVSRLFRLIKR
36	Ichthm4	SSLRSFRVLRPLRTIST	SVFRSVRILRIFRVLRVTRLVR	EILRLLRTLRPLRFVSH	VFRLLRVTRLLRLIKN
37	Ichthm5	EFFQILKLTRILTIFKV	LLCKSFKVFRIINFFL	EEYVSFAKFKALKILII	ISNR.6.KALQTLRLYRIIKH
38	Oxytrt1	RGLRTLRVLRPLRSINA	SGFRAVRLFRIFKLAR	KVIRMLRVLRPLRMISR	IVRAFRIGRVFRLIKK
39	Oxytrt2	KVIRTLRVVRPLRSIKA	SAFRAIRLLRVFKLAR	KSIRMLRVVRPLRVISR	LIRSIRIGRVLKIVSK
40	Oxytrt3	KSLRTLRVMRPLRSINA	SALRVFRVIRIFQLAK	KILRLLKVLRPLRVISR	IIRSFRIGRIFKLFRR
41	Oxytrt4	KSLRVMRVLRPLRSINA	TALRAFRLIRVFKLAK	KIFKIFRVVRGLRLVSK	IIRAFRITRIFRIVKR
42	Oxytrt5	KSLRVLRVLRPLRSINA	TALRAFRLIRIFKLAK	KTFKMLRVVRGIKLVSK	IIRAFRITRVFRIVKR
43	Oxytrt6	KALRTLRVLRPLRSVKA	SAFRAFRLLRVFKLAK	KAIRMLRVLRPLRIISR	FIRAFRISRIFRLIKR
44	Oxytrt7	KYLRTLRVIRPLRSVKA	LAFRAARLLRIFKLAK	KSIRLFRILRPLRVISK	FLRVFRLGRILRLIKR
45	Oxytrt8	KSLRVLRVLRPLRSINA	TALRAFRLLRIFKLAK	QVIKVLRVTRPLRLISR	IIRAFRISRVFRLVKK
46	Oxytrt9	SAIRVIRILRPLRTINS	TVLRAFRLLRIFKIVK	RGFRALRALRPLRMVSK	ALRIIRVARLLRMIKA
47	Oxytrt10	SGLRAFRVLRPLKSISS	SAFRSIRIFRTFRVLRVARLLR	KILRLLRTLRPLRFISH	VMRVMRVSRLFRLINK
48	Oxytrt11	SSLRVFRVLRPLRTITK	*Deletion*	KILRLLRTLRPLRLISK	VLRVLRVSRLFRLIGK
49	Oxytrt12	SGLRAFRVLRPLRAVSS	SAFKTVRIFRTFRVLRVARLLR	KILRLLRTLRPLRFISH	VLRVARVTRVIRLVGK
50	Oxytrt13	QVLRAFRVLRPLRTITS	SAFRAVRIFRTFRVLRVARLLR	KVLRLLRILRPLRFITH	ILRVLRVTRIVRLIGK
51	Paramt1	SAIRTIRILRPLRSIKS	SAFRTLRVFRIFKLAR	KILRIFRVLRPLRLVSR	VFRSFRIVRIMRLIRS
52	Paramt2	SAIRIVRIMRPLRSINS	QAFRTLRLFRLFKLAR	KILRVIRVLRPLRLIVR	ILRTFRIFRVLRLIKS
53	Paramt3	SAIRIIRIMRPLRSINS	QAFRTLRLFRLFKLAR	KILRVIRVLRPLRLIVR	ILRTFRIFRVLRLIKS
54	Paramt4	NSLRSFRVLRPLRTISS	SAFRAVRIFRAFRVLRVTKLMK	KILRLLRTLRPLRFITH	VFRVLRVTRLFRLVKQ
55	Paramt5	NALRSFRVLRPLRTVSS	SAFRSVRIFRAFRVLRVTKLMR	KILRLLRTLRPLRFITH	IFRVLRVTRLFRLIKS
56	Paramt6	NALRSFRVLRPLRTVSS	SAFRSVRIFRAFRVLRVTKLMR	KILRLLRTLRPLRFITH	IFRVLRVTRLFRLIKS
57	Paramt7	SALRSLRVLRPLRTISS	SVFKSVRIFRAFRVLRVTKLMR	KILRLLRTLRPLRLISQ	VFRVLRVTRLFRLVKS
58	Paramt8	NALRTIRILRSLDAGQS	SALRSLRIFRALRVLRISKLIR	KILRLFRTLRPLRFVSH	GFRILRAVRLLRLVKQ
59	Paramt9	NALRTIRILRSLDAGQS	SALRSLRIFRALRVLRISKLIR	KILRLFRTLRPLRFVSH	GFRILRAIRLLRLVKQ
60	Paramt10	SALRSLRVLRPIKAISS	QAFRSLRLFRAFRIFRVTKLVR	KVLRLLRTLRPLRLVKE	VFRVLRVTKLFRLVKQ
61	Paramt11	STARLLKILSVISVFSK	DLLNSSQALLIYRIIK	QYLKAIKYARPFRFLYL	ILRLAFACRNLRTLLIIQQ

№	Name	S4-D$_{I}$	S4-D$_{II}$	S4-D$_{III}$	S4-D$_{IV}$
62	Paramt12	HILRVIRIFRALRLIQD	NFVKALKMLRLMKFLY	QHPLS-KFITSLRILQF	YFKR.6.VAYQLQRNYRIVKR
63	Paramt13	TPLRMTRLLKHLGGIFA	RLMRSTKCLLFYRCIA	IVIETFIYFRPLKLLYR	LLRVLYTLRNLRIILIFQG
64	Paramt14	TPLRMMRLLKQLGAIFE	RLMRSTKCLLFYRCIL	EVIETFLFFRPLKLLYR	LLRVMYMLRNLRVIIIFQG
65	Paramt15	SPLRIITLLQYLGDIFD	RLIRGTKCLLFYRCLK	QILLYFGYFRPFNLLNR	LYRLCFALRSLRLILIFQG
66	Paramt16	STARLFRLLTVMSIFSK	DLLSSFQTFLVYRIIK	KYVKILKYTRPFRFLYL	FVRLAFALRNLRTLLIIQQ
67	Paramt17	SPLRAMRILIQLAKISS	KVLRASKAFMLYRVIK	KFSLYLIYLRPLMMINM	FYRILFCLRDIRILLIIQE
68	Paramt18	SPFRMLTLLNYLGDILK	RLIRGTKCLLFYRCLK	DVLMFLGYFRPMILMYR	LYRFFFALRSLRIILIFQG
69	Paramt19	SPLRAMRILIQLAKISS	KVLRASKAFMLYRVIK	KFSLYLIYLRPLMMINM	FYRILFCMRDIRILLIIQE
70	Paramt20	SPLRIITLLQYLGDIFD	RLIRGTKCILFYRCLK	QIFLYVGYFRPLNLLNR	LYRICFMLRSLRLILIFQG
71	Paramt21	YIFRALFLVDFIVQTKY	FVLNAFKGLRLLWFFN	GYLKAFRALRTLRVFSS	MF-M.6.RCLRVLNTYKILKL
72	Paramt22	DVIKTIRLLRLFRLIQE	TFFKSLKILRVFRFVY	HTVF--NFFSSLRIIEF	YLQR.6.VGFQLLRNYYIIKR
73	Paramt23	SPLRLVTLLMYLTNIFQ	RLIRGTKCLLFYRCLK	KILLYLGYFRPMKLMYR	LLRLFFSLRSLRIILIFQG
74	Paramt24	SPLRLVTLLMYLTNIFQ	RLIRGTKCLLFYRCLK	KILLYLGYFRPMKLMYR	LLRLFFSLRSLRIILIFQG
75	Paramt25	SPLRMITLLFYLGDIFV	RLIRGTKCLLFYRCLK	DLCLYLGYLRPMKLLFR	LLRLFFSLRSLRIILIFQG
76	Paramt26	SPLRLITLLLYLGDFVP	RLLRGTKILLFHRCLK	HLFLYLRYLRPFKLLYS	IIRLLFALRNLRILILFKG
77	Paramt27	SPLRLVTLLMYLTNIFQ	RLIRGTKCLLFYRCLK	KILLYLGYFRPMKLMYR	LLRLFFSLRSLRIILIFQG
78	Paramt28	SPLRMITLLFYLGDIFV	RLIRGTKCLLFYRCLK	DVCLYLGYLRPMKLLFR	LLRLFFSLRSLRIILIFQG
79	Paramt29	SPLRVITLLLYLGDIFA	RLIRGTKCLLFYRVLK	KFLLYFGYFRVMKLMYR	FLRLFFALRCLRIILIFQG
80	Paramt30	KILKSIRLLRVIRLPEE	QVFKAIKLIRVIKFSY	RNHVI-NFISSLRVFEF	YFSR.6.VAIQMLRNFRIIKR
81	Paramt31	SPLRLITLLLYLGDFVP	RLLRGTKILLFHRCLK	HLFLYLRYLRPFKLLYS	IIRLL---ILILFKG
82	Paramt32	STFRLIRIPFYVGKISS	TYIRILKSLFFFRVIK	EWMLFLGYLRPLKVTST	WLRLFILHRCLRMALIIQQ
83	Paramt33	TPLRMTRLLKHLGFIFA	KLMRSTKCLLFYRCIA	MVIETFLFFRPLKLLYR	LLRVLYTLRNLRVIIIFQK
84	Paramt34	SPLRIITILLYLGDGLL	RMIRGTKCILFYRCLK	DVCLFFGHFRPMKLMYR	ILRLAFMTRQLRLLGIFQG
85	Stylol1	KALRTLRVLRPLRSIKA	SAFRAFRLLRIFKLAK	KAIRLLRVLKPLRIISK	FIRAFRISRIFRLIKR
86	Stylol2	KSLRVLRVLRPLRSINA	TALRAFRLLRIFKLAK	KIIKIFRVARPLRLISR	IIRAFRISRVFRLIKK
87	Stylol3	SAIRVIRILRPLRTINS	TVLRAFRLLRIFKIVK	RGFRALRALRPLRMVSK	ALRIIRVARLLRMVKA
88	Stylol4	RGLRTLRVLRPLRSINA	SGFRAVRLFRIFKLAR	KVIRMLRVLRPLRMISR	IARAFRIGRVFRLIKK
89	Stylol5	*Deletion*	TALRAFRLIRVFKLAK	KTFKMLRVIRGIRLISR	IIRAFRITRIFRIVKR
90	Stylol6	KYLRTLRVIRPLRSIKA	IAFRAGRLLRIFKIAR	KSLRVFRILRPLRMISQ	FLRIFRLGRVLRLVKK
91	Stylol7	KSLRTLRVLRPLKSINA	*Deletion*	KILRLLKVLRPLRVISR	IIRSFRIGRIFKLFRR
92	Stylol8	QVLRAFRVLRPLRTITS	SAFKVVRIFRTFRVLRVARLLR	KILRLLRTIRPLRFITH	VLRVLRVTRIVRLIGK
93	Stylol10	SSLRVFRVLRPLRTITK	TAFRTVRIFRVLRVARLLR	KILRLLRTMRPLRLISK	VLRVLRISRLFRLIGK
94	Stylol111	SGLRAFRVLRPLKSISS	SAFRSIRIFRTFRVLRVARLLR	KILRLLRTLRPLRFISH	VMRVMRVSRLFRLINK
95	Tetrat1	TALRSLRVLRPLRSISK	SALRSIRILRVFRVLRVTRLLR	KILRLLRTLRPLRLLSQ	VLRVLRVSRLLKLIKS
96	Tetrat2	SALRSLRVLRPLRSISK	SAFRSLRILRVFRVLRVTRLLR	KILRLLRTLRPLRLLSQ	ILRVLRVSRLFKLMKS

№	Name	S4-D_I	S4-D_{II}	S4-D_{III}	S4-D_{IV}
97	Tetrat3	SAVRSLRVLRPLRTISR	SAFRSLRILRVFRVLRVTRLLR	KILRLLRTLRPLRVLHH	IFRVFRVTRLLRLIKQ
98	Tetrat4	SALRSLRVLRPLKSITK	SAFRAIRILRVFRVLRVTRLVR	KILRLLRTLRPLRLLNH	VLRVMRVSRLLKLIKS
99	Tetrat6	EILNIIKVFRVLFLVRT	IFIRTTKVLRIYRLIY	TIYVL-RIFKIFRLLLL	YFYKIF.5.SLQLIKVYRILAH
100	Tetrat7	LICRIFAMLRPMRILQM	HAIKSINVFRIFQVVK	KLAIL-NALRAFHIVVL	YFKLLEVFRILRLINT
101	Tetrat8	IILRVLGMLRPLKLIYH	RGLQTAKSIEILRIFQLIN	DYVIF-RTLRTLNLFHL	IHHR.6.EFISVFRVLRLAKT
102	Tetrat9	TVFSIIKFLRILTFFRL	ILIRCTKVFRIFKIFF	SNFVF-KCIAIFRVIFL	NYYR.6.KGLQLIRLIRVFNH
			STRAMENOPILES		
			Bacillariophyta		
103	Thalap1	SVLRSFRVLRPLRSISK	SIFRSLRLVRLFKMIK	RVLRTLRVLRPLKMIHR	LMSLIRIGRLLRLIRL
			Phaeophyta		
104	Ectocs1	TAIRVFRVLRPLKSISA	SALRSFRLFRVFKLAR	RSLRTLRALRPLRMINR	IIRTFRIGRVLRLVRG
			Raphidophyta		
105	Chatts1	GSLRVFRVVRPLRTISV	SALRFFRLCRLIKLVK	RALRAMRAIRPLRLIQR	TARVARVLLLFRVIRR
106	Chatts2	SALRLFRILRPLKTLSK	SALRSFRLFRVLKLAK	KTLRIIRVLRPLRMISR	VVRIFRLGRIFRLINS
107	Chatts3	SGLRTVRVLRPLKAISF	TALRIVRICRLGKLLRLVSITRIQR	RSIRLIRCIRPLQLLAR	FYKLMESLAALRVLRFIAL
108	Chatts4	GVLRLLRILRPLRTVKR	TVLRMVRMTRLMRVIRMAK	NVALTLKSLRPLRLGLR	VLRSLRILRIARLALY
109	Chatts5	SFLRALRSLRPLRSVKF	VILRAMSIFKLCRIMRFSK	KVLAVCASFRPMRLGIR	MLRGLRVLRLFRLCLL
110	Chatts6	SAFRTFRVLRPLKSLSK	SALRTFRLFRIFKLAK	MTLRVIRVIRPLRMVQR	VVRMCRIARVFRIANQ
			Oomycota		
111	Albugc1	SAIRTIRVLRPLRSLSM	SALRTFRLFRVFKLAR	RPLRGLRAFRPLRMISR	LIRVFRVTRIMRLIKA
112	Albugc2	LVLRTCRLLRPLRTLTN	SALRTLRLFRVLKLAG	RSLRALRALRPLRVIGR	ALRACRMGLAIRLMKR
113	Albugc3	LSLRLLRCIGHFRIWKG	LVLGLFRLVRPFRMIR	TIFKLGRALGPIRVFYR	MTRAFDFEYISLVFD---T
114	Albugl1	SAIRTIRVLRPLRSLSM	SALRTFRLFRVFKLAR	RPLRGLRAFRPLRMISR	LIRVFRVTRIMRLIKA
115	Albugl2	LALRTCRLLRPLRTLTN	SALRTLRLFRVLKLAG	RSLRTLRALRPLRVIGR	ALRACRMGLAIRLMKR
116	Albugl3	LSLRLLRCILHFRVWEG	FVLGLFRLIRPFRMKR	TIFKLGRALGPIRVFYR	MTRAFDFAYISLVFD---K
117	Aphana1	GYIRVLRVMRPLRTLHS	TAFRA---ARAFKLAR	RSLRAFRALRPLRVINK	VIRVFRVGRALRLIKK
118	Aphana2	ASLFRLLRVFKVLRGIRL	NIMFTCRVLRLLHLFR	KIFRLFRALRPLRAIHL	VLRILRLVRVYHLVSN
119	Aphana3	GFIRVLRVMRPLRTLHS	TAFRA---ARAFKLAR	RSLRAFRALRPLRVINK	VVRVFRVGRALRLIKK
120	Aphana4	GSIRVLRVLRPLRTLHS	TALRA---ARAIKLTR	QALRPARALRPLRVINK	LVRVARVSRTLRLIRQ
121	Aphana5	AQLRMLRILRPLRTLHS	ATFRL---IRALKLVR	RSLRSLRALRPLRVINK	AFRVFRVGRALRLINK
122	Aphana6	SAFRSFRALRSLRFFKG	FIFRMFRFLRPFRMLH	EYFHLGRAFGPVRVIRG	VLRAFDFNHLTLVLEPFPN
123	Aphana7	GFIRVLRVMRPLRTLHS	TAFRA----ARAFKLAR	RSLRAFRALRPLRVINN	VIRVFRVGRALRLIKK
124	Aphana8	SGIRCLRILRPLRTLRS	IALRA---LRAVKLAQ	RSFRCLRALRPLRVINQ	VVRVFRVGRALRLINK
125	Aphana9	GFIRVLRVMRPLRTLHS	TAFRA---ARAFKLAR	RSLRAFRALRPLRVINK	VIRVFRVGRALRLIKK
126	Aphani1	ASLFRLLRVFKVLRGIRL	HAMFVCRVLRLLQLFR	KVFRLFRALRPLRAIHL	VLRMLRLVRVTHLVSN

№	Name	S4-D$_{I}$	S4-D$_{II}$	S4-D$_{III}$	S4-D$_{IV}$
127	Aphani2	GSIRVLRVLRPLRTLHS	TALRA---SRAIKLTR	RALRPARALRPLRVINK	LVRVARVSRTLRLIRQ
128	Aphani4	SAFRSFRALRSLRFFKG	FIFRMFRFLRPFRMLH	EYFHLGRAFGPVRVIRG	VLRAFDFNHLTLVLEPFPN
129	Aphani5	TQLRMLRILRPLRTLHS	ATFRL---IRALKFVR	RSLRSLRALRPLRVINK	VFRIFRVGRALRLINK
130	Aphani6	SGIRCLRILRPLRTLRS	IALRA---LRAVKLAQ	RSFRCLRALRPLRVINQ	VVRVFRVGRALRLINK
131	Phytoi1	SAIRTIRVLRPLRSLSV	SALRSFRLFRVFKLAR	RSLRGLRTFRPLRMISR	LVRVFRVTRILRLVKA
132	Phytoi2	SMLRVFRVLRPLRSLNA	SGLRSFRLFALFKLAR	KTLRTFRALRPLRMINR	TVRSFRVGRLFRLVHS
133	Phytoi3	GSGNTWKVLRGLKTARSLTL	SSLRTLRVTRLMMK---	SVFRAGRVLRPLRMLNH	AGRIFRMMRVLRFVHL
134	Phytop1	SAIRTIRVLRPLRSLSV	SALRSFRLFRVFKLAR	RSLRGLRTFRPLRMISR	LVRVFRVTRILRLVKA
135	Phytop2	SVIRTIRVLRPLRSLSM	SSLRSFRLLRLFKLAR	RSLRAFRAFRPLRVISR	LARVFRVTRLIRLVKA
136	Phytop3	SMLRVFRVLRPLRSLNA	SGLRSFRLFALFKLAR	KTLRTFRALRPLRMINR	TVRSFRVGRLFRLVHS
137	Phytop4	GGGNTWKVLRGLKTARSLTL	SSLRTLRVTRLMMK---	SVFRAGRVLRPLRMLNH	AGRIFRMMRVLRFVHL
138	Phytop5	LSLRVFRGVRIFKRWKS	FVLRLFRLVRPFRVVR	SLFRLGRAFGPIRVFYR	MTRAFDFKHLSLVLE---R
139	Phytos1	SAIRTIRVLRPLRSLSV	SALRSFRLFRVFKLAR	RSLRGLRTFRPLRMISR	LVRVFRVTRILRLVKA
140	Phytos2	SELRTIRVLRPLRSLSA	SLLRAFRLFRIFELAR	LSLRDLRSLRPLRMISR	VVRLIRVARIFRLVES
141	Phytos3	SAIRTIRVLRPLRSLSV	SALRSFRLFRVFKLAR	RSLRGLRTFRPLRMISR	LVRVFRVTRILRLVKA
142	Phytos4	SMLRIFRVLRPLRSLNA	SGLRSFRLFALFKLAR	KTLRAFRALRPLRMINR	TVRSFRVGRLFRLVHS
143	Phytos5	SAIRTIRVLRPLRSLSM	SSLRSFRLFRVFKLAR	RSLRTFRAFRPLRVISR	LGQSFQGDSPHSDDAV…
144	Phytos6	AGMRALLVLRPLRFFNA	AGLRSMRIFRLFKLAR	SSLRTFRALRPLRMIHR	MIRGVRVLRLIRLIQT
145	Phytos7	SEFRAVRVLRPLRSLSA	SLLRAFRLVRVFRLAR	LSLRDLRSLRPLRMISR	VVRLIRVARIFRLVES
146	Phytos8	GGGGKWKVLRGLktvRSLTL	SSLRTLRVTRLMMK---	SVFRAGRVLRPLRMLNH	AGRIFRMMRVLRFVHL
147	Saprod1	GQLRILRVLRPLRTLRA	AALRA---LRAIKIAK	RALRSLRALRPLRVINK	IIRVFRVGRALRLINH
148	Saprod2	GFIRVLRVLRPLRTLHS	TSFRA---LRALKIAR	RALRGLRALRPLRVINK	VVRVFRVGRALRLIKQ
149	Saprod3	LGLFRLLRILKVLRGITF	RGFRIVRVARVFALGK	KPLRAFRALRPLRIAHH	VLRMLRVVRAYRLLKN
150	Saprod4	GTLSQWRVLRGLKAYRCFTA	SILRTLRIGRLMWR---	KVLRVGRVLRPLRLFNQ	VGRIFRVMRVMKFVKV
151	Saprod5	GQLRILRVLRPLRTLHS	AILRA---ARAIKLAY	RALRSLRALRPLRVIHR	VVRVFRVGRVLRLFQK
152	Saprod6	GILRVLRILRPLRALHS	TALRA---FRAIKIAR	RALRSLRALRPLRVINK	VVRVFRVGRALRLIKK
153	Saprod7	TGIRFLRILRPLRTVRS	TALRA---LRVLRLAQ	RALRTLRALRPLRVISQ	VLRVFRAGRALRLIQK
154	Saprop1	GFVRVLRVLRPLRTLHT	TAFRV---ARAFKLAR	RALRAFRAFRPLRVINK	VVRVFRVGRALRLIKK
155	Saprop2	LGLFRLLRILKVLRGITF	RGFRIVRIVRVFALGK	KPLRAFRALRPLRIAHH	VLRMLRVGRAYRLLKN
156	Saprop4	TGIRFLRILRPLRTVRS	TALRA---LRVLRLAQ	RALRTLRALRPLRVISQ	VLRVFRAGRALRLIQK
157	Saprop5	GTLSQWRVLRGLKAYRCFTA	SILRTLRIGRLMWR---	KVLRVGRVLRPLRLFNQ	VGRIFRVMRVMKFVKV
158	Saprop6	SAFRSFRALRSLRFFKG	FLFRIFRLLRPLSILR	DYFHLGRAFGPVRVIRG	VTRAFDFNHLTLVLELFPY
159	Saprop7	GQLRILRVLRPLRTLHS	AILRA---ARAIKLAY	RALRSLRALRPLRVIHR	VVRVFRVGRVLRLFQK
RHIZARIA					
Cercozoa					

№	Name	S4-D_{I}	S4-D_{II}	S4-D_{III}	S4-D_{IV}
160	Lothag1	SAIRVIRVLRPLRAVKR	SGLRTLRLLRIFKLFN	ESLKIFRALRPMRLLIR	ALRLFRLARLFRMFAK
161	Lothag2	TAIRAFRVLKPLRAIKS	STLRSFRVIRIFKLLR	KGFRALRALRAMRTITH	IFRVFRVLRIFRLMVK
162	Plasmb1	TSVRLLRLLRALRMVGV	IALRSFRLLRLMR---	ALSRALRAMRPLRMVKR	TLRVTRVFRVLRLFRH
'HACROBIA'					
Haptophyta					
163	Chryss1	SWLRAVRVLRALRTVNR	DVLRAMRVARVLRLLR	KALRAIRTLRPLRVIQR	MLRVFRIARLLRLMKM
164	Chryss2	SVLRAFRALRPLRALKR	SFLRILRMLRVARMLRLMR	ANLRVLRVLRPLRLVSR	TLRVLRIVRILRLLRS
165	Gephyo1	SIIRAARAMRPLRALKR	SFFRILRVARMFRLMR	STLKTLRALRPLRIAGR	MLRILRILRILRLLKK
166	Gephyo2	TWLKLFRTLRPLRAINR	SALRALRVLRMLKMLK	RALRAFRALRPLRLVAR	VVRLLRVVRVLRLLKR
167	Isochg1	SLIRTARALRPLRILKH	SFFRVLRVARMLRLMK	YAFRALRVLRPLRLVEK	VLRVLRILRILRLLKS
168	Isochg2	AFLKLFRILRPLRAVNR	SPLRALRVLRVLKMVR	RALRAFRAIRPLRLISR	VVRLLRVIRILRILKT
169	Isochg3	AVLRSVRILRPLRTVQR	SPLRTLRLARAFKLAR	NALRALRVLRPLRLVNR	YLRLLRLLRLVRVIQS
170	Isochg4	SVLRLFRMLRPLRALKR	SALRSFRALRILKLVR	KMLRVLRALRPLRLIAR	VLRLFRVVRILRILRT
Cryptophyta					
171	Guillt1	TVLRTARILRPLRTMTR	SALRGLRMLRLLKLAQ	KVLRALRALRPLRVIKR	PLRALRIVRVFRLVRR
172	Guillt2	TVLRVARILRPLRTMTR	SALRGLRILRLLRLAK	KVLRALRALRPLRVIKR	PLRALRIVRVFRLVRR
173	Guillt3	SFLRTVRVLRPLRTMTR	SALRAVRLFRLFKLAR	KSLRTLRAIRPLRVIKR	ALRTFRILRVFRLIRR
174	Guillt4	SALRSLRVLRPLRAVNK	SVLRAFRLVRLTKFLR	RIFRIFRALRPLRILSR	ILRIFRIFRILRAFRIFKT
175	Guillt5	KLVQIFRLTRPLRALRE	SLLRLIRLTRLIRALK	RVLRVLRPFRTLRFLNK	VLRIIRVFRTLRLVRR
176	Guillt6	NALILRPFRILRILRPLVK	MLVGA---IRIYRIMR	KQARLFRALRPIRILKR	FAKSFRLTRLVRLMAR
177	Guillt7	FALRLQGPSIRSFRLLRLLKYMMR	TFLAV---LRMYRLMI	KVFRLGRALRPLRLMKR	AARAARVARVLRLMKM
178	Guillt8	AVLRASRMLRPLRAINK	SVLRAFRLVRLVKFLR	RIVKGLRALRPLRILAQ	VVRVFRLFRTLRTLRILRS
179	Guillt9	SALRSLRLLRPLRAINR	SLLRALRLFRLVKLLS	QSLSLFKSLRAFRPLAR	SVRLIRLVRILRILRILRS
180	Guillt10	KSLRTLRALRVLRGIRR	TSLRALRLLRLVRVFR	RLLRVTKAMRPLRFVAK	MVRIFKIFKTLRAIKA
ARHAEPLASTIDA					
Chlorophyta					
181	Bathyp1	TTLRLMKCFQPLKALSK	SFLRVFRVFRILRTLKIVR	KTLRILRVLRPLRTIQR	IIRMVRFFRLLKVIQV
182	Bathyp2	GHLRVFRATLALRSFSF	VALRVVRLFDHPSAGIEKNSGKSTK…	RAINVLVSLRMFRVLSR	IGRPFRFLRTFRILRH
183	Chlamr1	TGIRTVRALRPLRTVNR	SVLRAFRLLRIFRLAR	RVLRSLRALRPLRAVNR	VLRVLRVVRVLRLVRT
184	Chlamr2	TALRTVRVLRPLRAITR	SVLRAFRLLRIFRLAR	RILRTLRALRPLRAASR	VLRVLRVVRVVRLIRR
185	Chlamr3	NAIRALRALRPLRTITR	RVLKALRVLRLFKLFR	SALRVLRAFKPLRLLTR	ILRVFRVQKLIALVRVARFA QLVKS
186	Chlamr4	SGIRAMRALRPLRALNA	QSLRTFRLLRILRSLKLLA	RTLRLLRVLRPLRMVSR	VFRIMRIGRMFKLIKN
187	Chlamr5	RALRALRALRPLRTVTR	AALRAFRALRLLKAFR	AALRALRALKPLRLLTR	ILRIFRLQRLL--RVMRLVRK
188	Chlamr6	NGLRALRAMRPLRALKA	QAFRTMRVLRVLRSLKLLR	KAVRLVRALRPLRLVKR	AMRLLRVCRMFKLIRG

№	Name	S4-D_I	S4-D_{II}	S4-D_{III}	S4-D_{IV}
189	Chlorv1	AAARSLRLLLALRSITL	SSLRAVRILRLLRLLT	EALRLLRVLRPLRLIAR	ALRAIRALRILRAVKH
190	Microp1	SALRVVRVLRPLRTLSI	TALRAFRIMRVLKLIR	RALRTLRALRPLRTIGR	MFRLFRLARVFRLIRT
191	Microp2	SAIRSARVLRPLRTITN	SVLRTFRLLRIFKLAR	RALRTFRALRPIRMASR	MIRIFRVARIFRLIPK
192	Microp3	SGMRTLRALKPLRTISV	TVLRAFRLVRVLRSIRILR	RSFRTLRALRPLRMVRR	ILRIFRMGRLFKILRG
193	Microp4	SALRTVRVLRPLRSITI	SVLRSARLLRILKLAR	RALRAFRALRPLRVAAR	LLRVFRIARVLRLIKR
194	Microp5	LALRAFRCFRPLRAMKS	ALLRMLRVLRIVAGLKSLR	RVLRVLRVLRPLRVIKN	VFSAARFFRLVKVLKS
195	Micros1	SGIRTVRILRPLRTITG	SVLRTFRLLRVFKLAR	RALRTFRALRPLRMASR	IIRVFRVARIFRLIPK
196	Micros2	SALRVIRVLRPLRTLSV	SALRSFRILRILKLVR	RALRTMRVLRPLRVISR	VFRILRLARIFRVVKK
197	Micros3	SSLRVIRVLRPLRAMSI	TALRAFRILRVLKLIR	RSLRTMRVLRPLRVISR	VLRVLRLARIFRLIRK
198	Micros4	LALRAFRCFRPLRAMKN	ALLRMLRVMRILAGLKGLR	KVFRVLRVLRPLRVIKN	VFRTARFFRLFKVLKS
199	Micros5	RALRALRALRPLRTITR	KVLKTFRVFRIFKMFR	RSLRILRAIRPLRALTK	LIRLFRVSRMFRLIKS
200	Micros6	SGIRTLRALKPLRTISG	SVLRAFRLVRVLRSVKFLR	RSFRILRALRPLRMVRR	LLRTFRLGRLFRLLKD
201	Micros7	SAIRTVRVLRPLRTLQG	TVLRSFRLLRILKLAR	RSMRSFRALRPMRMAAR	MLRVFRVARVLRLVRR
202	Micros8	NSLRAIRAVRPLRTLSR	GFIRALRVLRVVRTVRVLK	RSLRLLRILRPLRAIRR	VLRTFRFLRLFKIIKG
203	Monorn1	TALRAVRVLRPLRTVTK	SVLRTFRLLRVFKLAR	RALRTLRALRPLRAASR	LLRMLRVARMFRLIPR
204	Ostrel1	STLRTVRILRPLRTISM	TVLRALRVFRILKLMR	RALRTLRVLRPLRLLGR	AFRALRICRVFRMVKK
205	Ostrel2	GQLRFFRGVHALKRFKL	TSLRIPRLFKTFGPAK---	RAFNMAAPMRPIRIMTR	IGRPFRFLRVFRIIRH
206	Ostrel3	VTVRLLRSLYAFKLFAK	SFIRLLRIGRALRTFRVIR	RALRLLRIMRPLRSIRR	YLQLVRLLRLLKLVKA
207	Ostret1	STLRAIRIIRPLRTISM	TVLRALRVFRILKLVR	RALRTLRVLRPLRLIGR	AFRALRICRVFRMFKQ
208	Ostret2	TAVRGLRALRPLKSISS	AVARAFRLLRVLRATRLIAR	RTFRLVRVLRPLRLLNR	VLRVVRLGRILRLFKQ
209	Ostret3	GQLRLFRGVHALKRFKL	TSLRIPRLFKTFGAAK---	RAFNMAAPVRPVRIMTR	IGRPFRFLRVFRVIRH
210	Ostret4	GTLRILRALQPFRALS-	SFLRIMRILRLFRVARVFR	RSLRTVRILRPLRSVNR	MFRMLRFLRLFKVVQV
211	Ostret5	TAVRGLRALRPLKSISS	AVARAFRLLRVLRATRLIAR	RTFRLVRVLRPLRLLNR	VLRVVRLGRILRLFKQ
212	Volvoc1	TAIRAFRAMRPLRTINR	SVLRAFRLLRIFRLAR	RVLRALRALRPLRAVQR	VMRVLRVVRVVRLVRQ
213	Volvoc2	TAVRTVRVLRPLRAITR	SVLRMLRLLRIFRLMR	RVLRTLRALRPLRAASR	VMRVLRVVRVVRLIRR
214	Volvoc3	TGLRTIRALRPLRTVKR	SVLRALRLLRIFRLAR	RVLRTLRALRPLRAVQR	LLRVLRVARV------
215	Volvoc4	RALRAVRALRPLRTVTR	AALRAVRALRLLKAFR	SALRVLRALKPLRLLTG	VLRVFRLQRLL--RVARLLRQ
EXCAVATA					
Euglenozoa, Kinetoplastida					
216	Leishb1	TAWRLIRAIKCL-----	SLFNWVRFLRLFRVIP	KVMRCFRILGPLRYWKW	VLRLLRVGRVLHLIHL
217	Leishi1	TAWRLIRTIKCL-----	SLFNWVRFLRLFRVIP	KVMRCFRILGPLRYWKW	VLRMLRVGRVLHLVHL
218	Leishm1	TAWRLIRTIKCL-----	SLFNWVRFLRLFRVIR	KVMRCFRVLGPLRYWKW	VLRMLRVGRVLHLVHL
219	Leptos1	TAWRLVRTIKCF-----	AVFNWVRFLRLFRVIS	KVMRCFRILGPLRYWRW	VLRMLRVGRILHLLNL
220	Leptos2	RWMRAFAAARPLRFFVF	RMVGSLRVLRYLHAAK	APLRVGRVIRTVRLCTT	TLRFLRVALLVKLIK-
221	Phytosp1	TAWRLIRCIKCL-----	SLFNWIRFLKLLRVCP	KVFRCFRIIGSLRYWTW	VLRLLRVTRVLRLVNF

№	Name	S4-D$_{I}$	S4-D$_{II}$	S4-D$_{III}$	S4-D$_{IV}$
222	Trypab1	SAFRLIRVLKSS-----	TVLNWVRLLRLFRTLP	KVLRCFRILIPMRVSNF	VLRMLRLGRFFSAAKV
223	Trypac1	TAFRLIRIIKAC-----	TVLNWVRFLRLFRIAP	KVMRCFRILGPMRYCRF	TLRLLHFGRMLKLVDF
224	Trypac2	TAFRLIRIIKAC-----	TVLNWVRFLRLFRIAP	KVMRCFRILGPMRYCRF	TLRLLHLGRMLKLVDF
225	Trypag1	TAFRLIRVIKSC-----	TVLNWVQFLRLLRVAP	KAMRCFRILGPMRHCTF	VLRLLHFGRLLTLMEF
226	Trypag2	RWMRAFAVARPLRFLLR	HVVRGLRTLRVLRLGK	APCRVGKSIQAIRLLTS	VVSFLRVIRVVKLLK-
227	Trypar1	TAFRLIRIIKAC-----	TVLTWVRFLRLLRIAP	KVMRCFRILGPLRHCSF	VLRLLHFGRMLNLVDF
228	Trypav1	TAFMLIRVIKAC-----	TVLNWVRFLRLFRVST	KVLRCFRVFMPLRHCKF	VLRMLRLGRLLRLVDL
**OBAZOA**					
APUSOZOA					
Apusomonadida					
229	Thecat1	SAIRTFRVLRALRTLSG	SVLRAFRLLRVFKLAR	RAIRTLRAFRPLRAITR	ILRVFRVARIFRIIKS
230	Thecat2	SSIRTIRILRPLRTISS	TVLRCFRLLRVFRLVR	RLFRALRALRPLKLIVR	VVRIVRLGRLLRLGKR
231	Thecat3	TAIRGLRVLRTLRTLRH	SVFRALRVARIFRLAR	RFLRGLRFLRLVRVLSR	VFRMFRLLRTVRLVAR
232	Thecat4	SAFRAFRALRPMRALKF	SFLRVFRVLRITRLLV	RLVRYFRALRPLRIVSR	GRRFFRIARIFRLVRH
233	Thecat5	TALRTLRVFRPLRTLRT	SVFRVVRLLRVVRILR	RGLRGFRALRPLRLVIR	TVRVLRVGRLFRLAKR
234	Thecat6	APFWVLRALRPLQSVSR	PRARMLRPLRVFKLTR	----ALRALRPLRLVSR	LIGSLQLLRFFQIVPL
235	Thecat7	SAIRTFRVLRALRTLSG	SVLRAFRLLRVFKLAR	RAIRTLRAFRPLRAITR	ILRVFRVARIFRIIKS
OPISTHOKONTA					
Metazoa (Na$_v$1)					
236	AplyNav1	QALRTFRVLRALKTISV	SVLRAFRLLRVFKLAK	KSMRTLRALRPLRAVSR	VIRVFRVGRVLRLVKS
237	BranNav1	SALRTFRVLRALKTISV	SVLRSFRLLRVFKLAK	KSLRTLRALRPLRAISR	VIRVARIGRILRLIKG
238	DrosNav1	AALRTFRVLRALKTVAI	SVLRSFRLLRVFKLAK	KTMRTLRALRPLRAMSR	VVRVAKVGRVLRLVKG
239	HaloNav1	AALRAFRVLRALKAISA	SVLRTFRLMRVFKLAK	RSLRTLRALRPLRAMSR	IIRLARIGRILRLIRG
240	HomoNav1	SALRTFRVLRALKTISV	SVLRSFRLLRVFKLAK	KSLRTLRALRPLRALSR	VIRLARIGRILRLIKG
241	MusmNav1	SALRTFRVLRALKTITV	SVLRSFRLLRVFKLAK	KSLRTLRALRPLRALSR	VIRLARIGRVLRLIRG
Metazoa (Na$_v$2)					
242	BranNav2	SGLRTFRILRALKTVSI	SVLRSFRLARVTRVLKLAK	RSLRVFRALRPLRAISR	VVRIFRIGRVLRLIRA
243	CyanNav	SGVKTFRVLRALKTIST	SVMRVFRLLRVLKLAQ	RSL---RALRPLRAVSR	VIRVFRLGRLLRFFDG
244	DrosNav2	AGLRTFRVLRALKTVSI	SVLRGLRLLRVLKLAQ	RSLRTLRALRPLRAISR	VVRVFRIGRILRLIKA
245	ExaiNav	SGIRIFRVLRALRTISA	SVLRTFRLLRVFKLAQ	RSLRTLRALRPLRAISR	VARVFRIGRLLRFYKG
246	HaloNav2	SGLRTFRVLRAFKSFTI	SVLRTLRLMRVFRLAR	RALRTLRALRPLRAISR	VVRVFRVFRVLRVIRA
247	MnemNav	SFIKALRVLRSLKVITV	SGLRSLRVLRVFRLAK	KAIRALRALRPLRAISR	CFRVFRVARILRIIQM
248	NemaNav2	SGIRTFRVLRALRTISA	SVLRTFRLLRVFKLAQ	RSLRTLRALRPLRAISR	VARVFRIGRLLRFYKG
249	PolyNav	SGIRTFRVLRALKTIST	AVLKVFRLMRVLKLAQ	RSL---RGLRPLRAISR	VVRVFRLGRLLRFFEG
250	StroNav2	NFLRTFRVLRALKTISV	SVLRSFRLLRVLKLAQ	RALRTLRALRPLRAISR	VLRLFRIGRVLRLVKQ

№	Name	S4-D$_{I}$	S4-D$_{II}$	S4-D$_{III}$	S4-D$_{IV}$
251	TricNav2	PGASVIRVLRALRMITA	SVLRTLRLLRVFKLAR	RSLRVLRALRPLRAISH	TLRLFRIVRILRVLEF
	Choanoflagellata (Na$_v$)				
252	MonoNav	AAIRTLRVFRALRSITA	SILRSFRLLRVLKLAR	RSLRTLRALRPLRAISR	VLRLLRVIRVLRVVKQ
	Metazoa (HVA Ca$_v$: Ca$_v$1)				
253	CyanCavL	KALRAFRVLRPLRLVSG	SVLRCVRLLRIFKVTR	RILRVLRVLRPLRAINR	FFRLFRALRLVKLLSQ
254	DrosCavL	KALRAFRVLRPLRLVSG	SVLRCVRLLRVFKVTK	KILRVLRVLRPLRAINR	FFRLFRVMRLVKLLSK
255	HaloCavL	RSLRAFRVLRPLRLVSG	SVLRCVRLLRIFKMTS	KILRVLPSVLRPLRAINR	FFRLFRVLRLVKLLSR
256	HomoCavL	KALRAFRVLRPLRLVSG	SVLRCVRLLRIFKITR	KILRVLRVLRPLRAINR	FFRLFRVMRLVKLLSR
257	StylCavL	KALRAFRVLRPLRLVSG	SVLRCIRLLRIFKVTR	RILRVLRVLRPLRAINR	FFRLFRALRLVKLLNQ
	Metazoa (HVA Ca$_v$: Ca$_v$2)				
258	ApisCav2	RTLRAIRVLRPLKLVSG	SVLRALRLLRIFKVTK	KSLRVLRVLRPLKTIKR	FLRLFRAARLIKLLRQ
259	CaenCav2	RTLRAVRVLRPLKLVSG	SVMRALRLLRIFKLTS	KSLRVLRVLRPLKTIKR	FLRLFRAARLIRLLQQ
260	HomoCavN	RTLRAVRVLRPLKLVSG	SVLRALRLLRIFKVTK	KSLRVLRVLRPLKTIKR	FLRLFRAARLIKLLRQ
261	HomCavPQ	RTLRAVRVLRPLKLVSG	SVLRALRLLRIFKVTK	KSLRVLRVLRPLKTIKR	FLRLFRAARLIKLLRQ
262	HomoCavR	RTLRAVRVLRPLKLVSG	SVLRALRLLRIFKITK	KSLRVLRVLRPLKTIKR	FLKLFRAARLIKLLRQ
263	SchiCav2	PAVRALRVLRPLKLVTG	SVLRALRLLRIFKVTR	KSLRVLRVLRPLKTINR	FLRLFRAARLIKLLRQ
	Choanoflagellata (HVA Ca$_v$)				
264	SalpCavL	KALRALRVLRPLRLITS	SALRSLRLLRIFREMKQ	RVLRVFRVLRPLRAIKR	FLRLFRVARLLKLVSR
	Metazoa (LVA Ca$_v$)				
265	CaenCavT	TAIRTVRVLRPLRAVNR	SVLRTFRLLRILKLVR	RVLRLLRALRPLRVINR	VMRVLRIARVLKLLKM
266	DrosCavT	TAIRTIRVLRPLRAINR	SVLRTFRLLRILKLVR	RVFRLLRSLRPLRVINR	VMRVLRIARVLKLLKM
267	HomoCavT	SAIRTVRVLRPLRAINR	SVLRTFRLLRVLKLVR	RVLRLLRTLRPLRVISR	IMRVLRIARVLKLLKM
268	LymnCavT	SAIRTIRVLRPLRAINR	SVLRTFRLLRILKLVR	RVFRLLRTLRPLRVISR	VMRVLRIARVLKLLKM
	Metazoa (NALCN)				
269	CaeNALCN	FPFLHLNYRAWYGAIRSIRPFIIIRLIPL	TYFQTFRLLRLIKA--	QFLMICRAMRPLRIYTL	FVALPSSKIDVDVQVE…
270	DroNALCN	SYLSIMRAPRPLIMIRFLRV	TYFQVLRVVRLIKA--	QLLMILRCVRPLRIFTL	ILRNDLSYFFGFMVV…
271	HomNALCN	SPWGMLRIPRPLIMIRAFRI	TYFQVLRVVRLIKI--	QLLMVLRCLRPLRIFKL	ALLNAYTYMMGACVI…
272	NemNALCN	IIFSIPRAPRALIMVRVFKL	AIFHVMRVLRLIGA--	QMLMIFRCLRPLRVLTL	SLKGNEKAYTVATIFGV…
	Fungi (Cch)				
273	AspeCch	YVFSMLSCLRILRLLNL	DVLTLFQILRVYRVVL	RAIGAFKALRALRLLNV	LNKLFLVSITLLIIPR
274	PeniCch	YIFRMLSCLRILRLLAL	TVLTLFQILRVYRFVL	RAIGAFKALRALRLLNV	LNKLFLVSIALLLIPR
275	PseuCch	YIFRALSVLRCARLLTA	AWLTFFQLARFYRVIA	RFTRALKAFRALRLINL	LQKLFLVCVALKLVQK
276	SaccCch	KSYDTKTGIRIFKPLailRILRL	AWLSIFHISRFYRVII	RIFKGLTALRALRCLTI	NIKGFFLLVIFLFIIPQ
277	TrichCch	YLFRALSTLRTSRLLVV	SWLTAFHLMRWYRVIL	RFTRSLKAFRALRLITL	VQKLFLVAFALKLVQR

Table S2. Transmembrane segments S4 in the analysed sequences of four-domain voltage-gated cation channels.
Sequence names shown in red colour correspond to channels with at least one arginine/lysine-poor segment S4, i.e. S4 bearing less than three positive charges (arginine (R), lysine (K), and histidine (H)) or S4 with positive charges separated by an extended sequence of hydrophobic amino acid residues. *Not analysed* - motif unidentified due to incomplete sequence. "-" – deletion; "_" – insertion.

№	Name	IG XXXX	DS A/F/N	pre-IQ motif xXxxXX+xxXxX	IQ motif I(L,V)Qxxx+xxxx+
**SAR**					
ALVEOLATA					
Dinoflagellata					
1	At406917	VLLT	A/Q/N	VASALAKRLFED	IQNSYHAFKFR
2	Ac156993	TCLT	T/M/N	STQQILRFSCIG	VQQAFHHYQKR
3	Ac29873	YLMT	T/R/N	ALRGLIRRICQ	IQRRYKERMLM
4	Kb20887	VMLT	T/M/N	AVKFVFRLSIAK	IQRFYQERKVE
5	Kb269344	FLLT	T/M/N	AVKFVFRLSIAK	IQKKFKEGRLN
6	Kb53452	SLLT	T/T/N	AVRMAVAVTLAH	IQAMFRTMKAW
7	Kb269973	LVLT	S/G/N	TLWRLASMVDGE	IQRVWRSTLAR
8	Kb20579	EMSS	A/A/N	ILMICTKRVYCW	VEKYFRHQLK-
9	Kf405392	YLLT	T/T/N	VVRQVFRLVTV-	*Not identified*
10	Kf407651	DIIS	A/A/N	ATRQVLRFATI-	VQRVVRAAIKK
11	Kf416989	FMLT	T/L/N	ATRQVLRFVAV-	LQRLFRARKAR
12	Lp90575	QTVS	T/M/N	ATRQVLKLTCTS	IQPRVRQVQAA
13	Om66755	IWAT	T/T/N	AILGAMRAIAAR	LQSAFRSRRD-
14	Pm40145	LKLT	T/M/Y	AAKQVMRYES--	IQYYFNQARPA
15	Pm47759	GMLT	T/-/Q	AAKMVLRLNLVE	VQRKFRE----
16	Pm20998	IMST	T/M/N	AAQSAIRIAC--	LQEVWHNRQKP
17	Pm2595	IMST	T/M/N	AAQSAIRIAC--	LQEVWHNRQKP
18	St17126	DLLD	G/A/N	VMMICAQRSYLW	VMRFVKK----
19	St9808	DIIS	A/M/N	AAKQVVRFASI-	IQMRIREVVLR
20	St26908	VLLS	T/S/N	AVRQVLRFVAV-	LQKVWK-----
21	St388392	LMLT	T/M/N	AVKQVLRITVAL	*Not identified*
22	St391309	LMIT	T/Y/N	*Not present*	*Not analysed*
23	Ssp188361	ILLT	T/A/N	ALLQVLKLLC--	IQRILRDRRAA
24	Ssp190870	VMLT	T/M/N	ACKQVMRFVC--	LQSAFKGVLRG
Perkinsozoa					
25	Perkim1	LFLT	T/I/N	*Not analysed*	*Not analysed*
26	Perkim2	LFLT	T/I/N	*Not identified*	LEAVAFVNEAK
Apicomplexa					
27	Toxopg1	SLLS	S/T/N	AVLNSAKRACEFH	IQNWFRKRHMVI
28	Toxopg2	SLLS	S/T/N	AVLNSAKRACEFH	IQNWFRKRHMVI
Chromerida					
29	Vitreb1	SFLS	S/Q/N	VLWSLASCVSGA	VQALWKGARKR
30	Vitreb2	PFMT	S/Q/N	VIINVVRRACTY	IQLKWEKRKKR
31	Vitreb3	VLLT	T/Q/N	AALAVLRRLIGQ	IQVCWRGWKDK
32	Vitreb4	LLIS	S/Q/N	VVINLVDRSAHW	IQSLYRK----
Ciliata					
33	Ichthm1	DILT	S/N/N	TLIQLSHKIVGQ	IERNLK----
34	Ichthm2	KELN	S/Q/N	LMYYLLRFSLLE	*Deletion*
35	Ichthm3	AFLT	A/Q/N	LMFFIQKYALNN	*Deletion*
36	Ichthm4	KYLT	A/Q/N	LMFFVQKFALQD	*Deletion*
37	Ichthm5	DKLQ	A/N/S	VVTKVSKISVTI	LQKKMRIWRRK
38	Oxytrt1	DALS	S/N/N	ILSCLLKKYISH	IQERIKPFLMK
39	Oxytrt2	HLLT	S/N/N	ILQSLSKAALQI	ARHQLKITRQK
40	Oxytrt3	FLLT	S/Q/N	VFESLALLMIVK	*Not identified*
41	Oxytrt4	NLLT	A/N/N	VLEALALRLVVQ	AVQHMKYVVAK
42	Oxytrt5	NLLT	A/N/N	VLEGLALRLVVL	SVKHMKFIVQK
43	Oxytrt6	FLLT	A/E/N	VLQSLTKILIVK	YQAMQRLKVR-
44	Oxytrt7	YLIS	C/M/N	VLNHLCRSIFKV	QQNTFRNNQSI
45	Oxytrt8	NLLT	A/N/N	VLDALSLRLMVH	AFNHMRYVVAK
46	Oxytrt9	HKFS	S/K/N	VMWAIFHSIIGN	LQHDNNNNVHS

№	Name	IG XXXX	DS A/F/N	pre-IQ motif xXxxXX+xxXxX	IQ motif I(L,V)Qxxx+xxxxx+
47	Oxytrt10	LMLK	A/Q/N	LLFRAMRRVYGE	*Deletion*
48	Oxytrt11	*Not identified*	C/Q/N	LLYRSMKRIYGD	*Deletion*
49	Oxytrt12	*Not identified*	A/Q/N	LLYRCMKRIYGQ	*Deletion*
50	Oxytrt13	*Not identified*	A/S/N	MLYRCMRRVYGN	*Deletion*
51	Paramt1	FYMT	A/E/N	VLEVLSHNILTR	IMRRMKWKLDE
52	Paramt2	KDLT	A/G/S	ALICAAQDFLES	IQQNTRRKFQR
53	Paramt3	RDLS	A/G/S	ALICAAQDFLES	IQQNAKRSLQK
54	Paramt4	QNLT	A/E/N	VLFAVLKNYLQK	*Deletion*
55	Paramt5	KILT	A/E/N	ILFCVMKSYMWK	*Deletion*
56	Paramt6	KILT	A/E/N	ILFSVMKSYMWK	*Deletion*
57	Paramt7	KNVS	T/D/N	VLFAFMKNFQTD	*Deletion*
58	Paramt8	SSLT	A/D/N	VLFAVMKEAYLE	*Deletion*
59	Paramt9	SSLT	A/D/N	VLFAVIKEAYLE	*Deletion*
60	Paramt10	KVLS	T/D/E	VFFTIMKMFQQQ	*Deletion*
61	Paramt11	QILT	A/R/Q	*Not identified*	LLMIFRTFKSY
62	Paramt12	HLLD	S/S/S	VILSLTKISVTL	IQKKFRLWKKR
63	Paramt13	QQLT	A/R/K	*Not identified*	SLVAFKVWRSY
64	Paramt14	QQLT	A/R/K	*Not identified*	ALTTFRVWKSY
65	Paramt15	GEFN	A/R/K	*Not identified*	VLIAFKAWESH
66	Paramt16	QILT	A/R/Q	*Not identified*	LLMIYRTLRTY
67	Paramt17	KFLT	A/R/K	*Not identified*	VLIALKTWEAY
68	Paramt18	NELS	A/R/K	*Not identified*	VLILFKTWQQF
69	Paramt19	KYLT	A/R/K	*Not identified*	VLIALKTWEAY
70	Paramt20	KDFN	A/R/K	*Not identified*	VLIAFKAWESH
71	Paramt21	KSLN	S/D/S	*Not identified*	*Not identified*
72	Paramt22	--LD	A/Y/S	VIVSLTRLSVEL	LQGKARMLINK
73	Paramt23	TEVT	A/N/K	*Not identified*	VLILFKTWESY
74	Paramt24	TEVT	A/N/K	*Not identified*	VLILFKTWESY
75	Paramt25	TDES	A/R/K	*Not identified*	VLILFKTWESY
76	Paramt26	DDLT	S/R/K	*Not identified*	ILVIFKAWQNY
77	Paramt27	TEVT	A/N/K	*Not identified*	VLILFKTWESY
78	Paramt28	TDET	A/R/K	*Not identified*	VLILFKTWESY
79	Paramt29	SDIT	A/R/K	*Not identified*	VLILYKAWEAY
80	Paramt30	FELE	A/H/S	VIVSLTKISVEL	LQNKARTLINK
81	Paramt31	DDLT	S/R/K	*Not identified*	ILVIFKAWQNY
82	Paramt32	PDLT	A/N/R	*Not identified*	LYMTIKAWKAF
83	Paramt33	QQLT	A/R/-	*Not identified*	TLITFKLWKSY
84	Paramt34	QNVT	A/R/K	*Not identified*	VLILFKTWQSY
85	Stylol1	FLLT	A/E/N	VLQSLTKILMVK	YQALQRLKQTVR
86	Stylol2	NLLT	A/N/N	VLDALSLRLMVK	AFNHMKYVVAK
87	Stylol3	HKFS	S/K/N	VMWAIFHSIIGN	*Deletion*
88	Stylol4	DVLK	S/N/N	ILSCLQKRYLQH	IQQRIKPFLNR
89	Stylol5	NLLT	A/N/N	VLEALALRLVVI	SVKHMKFIIQK
90	Stylol6	YILT	C/I/N	ILIHLVKIIFKI	QQSSIKNTLSN
91	Stylol7	FLLT	S/Q/N	VFESLALLMIVK	*Not identified*
92	Stylol8	*Not identified*	A/S/N	MLYRCMRRVYGN	*Deletion*
93	Stylol10	*Deletion*	C/Q/N	LLYRSMKRVYGD	*Deletion*
94	Stylol111	LMLK	A/Q/N	LLYRTMRRYYGE	*Deletion*
95	Tetrat1	AFLT	A/Q/N	LFYQLFKFSLYS	*Deletion*
96	Tetrat2	AYLT	A/Q/N	LLYQFMKFSLYE	*Deletion*

№	Name	IG XXXX	DS A/F/N	pre-IQ motif xXxxXX+xxXxX	IQ motif I(L,V)Qxxx+xxxx+
97	Tetrat3	KYLS	T/S/N	LLYYFFKHSLYD	*Deletion*
98	Tetrat4	KYLT	A/Q/N	LFFYFFKFSLKD	*Deletion*
99	Tetrat6	DKLT	V/G/N	VIIKLAEISLKI	IIRKIKEWKKR
100	Tetrat7	EKIT	S/S/P	VVVKMTQISLFL	IQRKIKQWMRT
101	Tetrat8	QHID	S/N/P	VVIQMTQVSLFL	IQRRIKNWMER
102	Tetrat9	HSLT	L/G/N	VVEKLSKISVQI	LSRKAKIWKRQ
STRAMENOPILES					
Bacillariophyta					
103	Thalap1	GLMT	A/N/N	VATLLAKRLTKQ	IIMAVRRF---
Phaeophyta					
104	Ectocs1	VLVT	A/N/N	VLLALSEEVHRL	IQKAFRRFLE-
Raphidophyta					
105	Chatts1	GLMT	T/D/N	*Not identified*	IQRIVRHFLFK
106	Chatts2	LFMT	V/D/N	VAVALAKRVVIK	*Deletion*
107	Chatts3	*Not identified*	A/H/N	VLYSQIILWRQE	*Not identified*
108	Chatts4	*Not identified*	T/K/T	LLEYGEVLRLMY	LQVACSKSKTV
109	Chatts5	MEMT	S/R/C	LLLACYGLTIY-	*Not identified*
110	Chatts6	IFMT	V/M/N	LATALARNYIMK	*Deletion*
Oomycota					
111	Albugc1	FMLT	A/T/N	VLLAMTRELVKE	IQHAVLRWLH-
112	Albugc2	IYTN	A/T/N	*Not identified*	IQSAVRRLLAT
113	Albugc3	LLLT	A/E/K	LLLVLTERIVPL	IQSVFRMYRVR
114	Albugl1	FMLT	A/T/N	VLLAMTRELVKE	IQHAVLRWLR-
115	Albugl2	AFTN	A/T/N	*Not identified*	IQSAVRRLLAK
116	Albugl3	LLLT	A/E/K	LLLVVTERIVPL	IQAVFRMYRVR
117	Aphana1	SMVT	T/D/N	VPRALVQRVLAE	IQRFLERVR--
118	Aphana2	LFIT	A/K/N	IFYTLCHHAMPN	IQEAWKAYLSR
119	Aphana3	AIVT	T/D/N	VPRALVQRVLAE	IHRFIARLR--
120	Aphana4	ALVT	V/D/N	VPRALVQRIMGD	LTRFVRNTRAR
121	Aphana5	VLVT	T/N/N	VPRALVQRTLSQ	IQNFLKSASYH
122	Aphana6	ILLS	A/Q/K	LLLILTQRTVPC	IQKTFRGSLVA
123	Aphana7	SLVT	T/D/N	VPRALVQRVLAE	IQRFFEKVK--
124	Aphana8	GFLT	T/D/N	VPRALALRTVSR	IAGWHRRRKR-
125	Aphana9	SMVT	T/D/N	VPRALVQRVLAE	IQRFLGKAR--
126	Aphani1	LFIT	A/K/N	IFYTLCHHAMPN	IQEAWKAYVAK
127	Aphani2	ALVT	V/D/N	VPRALVQRIMGE	IARFVRNKRAR
128	Aphani4	ILLS	A/Q/K	LLLILTQRTVPC	IQKTFRGSLVA
129	Aphani5	VMVT	T/N/N	VPRALVQRVLSQ	IITFLRKVNHH
130	Aphani6	GFLT	T/D/N	*Not analysed*	*Not analysed*
131	Phytoi1	FMLT	A/L/N	VLLAMTREMVKV	IQRQVAEWLR-
132	Phytoi2	FHLS	S/N/N	VARRVGKFVIDE	IHRSVNALIFR
133	Phytoi3	EILT	A/Q/Q	LLRILC------	*Not identified*
134	Phytop1	FMLT	A/L/N	VLLAMTREMVKV	IQRQVAEWLR-
135	Phytop2	FMLT	A/T/N	TLLAMTREMVKD	IQRSVATWLQ-
136	Phytop3	FHLS	S/N/N	VARRVGKFVIDE	IHRSVNALIFR
137	Phytop4	EILT	A/Q/Q	LLRILC------	VDSYVKGYIQR
138	Phytop5	LLLT	A/R/K	LLLVLTMRVLPL	IQSSFRMFRVR
139	Phytos1	FMLT	A/L/N	VLLAMTREMVKL	IQRQVAEWLR-
140	Phytos2	FLLT	A/S/N	VMLAMTREMMNK	IQRAVAAWLR-
141	Phytos3	FMLT	A/L/N	VLLAMTREMVKL	IQRQVAEWLR-
142	Phytos4	YLLS	S/N/N	VTRRIGKFVIDE	IHRSVNALVFR
143	Phytos5	FMLS	A/-/A	TLLAMTREMVKD	IQRAVAAWLR-

№	Name	IG XXXX	DS A/F/N	pre-IQ motif xXxxXX+xxXxX	IQ motif I(L,V)Qxxx+xxxx+
144	Phytos6	RLLS	S/N/N	VVRKLGKFVLDI	LHDAVRSLIFR
145	Phytos7	FLLT	A/S/N	VILAMTREMINV	LQRRVSNWLAR
146	Phytos8	EILT	A/Q/Q	LLRILC------	VDSYVKGYIQR
147	Saprod1	VLVT	T/D/N	VPRALVLQAVSS	IARTLLDHVHAR
148	Saprod2	AVVT	T/D/N	VPRALVTRVLAE	IQRFLTKSK--
149	Saprod3	IFLT	A/H/N	IFYALCHRTMPN	IQKAWHKH--
150	Saprod4	QILT	S/R/N	LLRTVC------	VDSYVKGYIQR
151	Saprod5	VMVT	T/D/N	VPRALVQRSLSE	IAKFLRRVRYR
152	Saprod6	TLIT	T/D/N	VPRALVQRILSE	IARFIYRYRQA
153	Saprod7	GFVT	T/D/N	VPRALVIRSMSE	ILRWLLRKVGAR
154	Saprop1	AVVT	T/D/N	VPRALVMRVLAE	IQRFVQRAR--
155	Saprop2	IFLT	A/H/N	IFYALCHRTMPN	IQKVWHKH--
156	Saprop4	GFVT	T/D/N	VPRALVIRSMSE	ILRWLMHKVGAR
157	Saprop5	xxLT	S/R/N	LLRTVC------	VDSYVKGYIQR
158	Saprop6	IFLS	A/Q/K	LLLVLTQRNVPG	IQKVFRGSLVA
159	Saprop7	VMVT	T/D/N	VPRALVQRSLSE	VAKFLRCVRYR
RHIZARIA					
Cercozoa					
160	Lothag1	AFLT	A/E/N	*Not identified*	VSSPRNNLDTK
161	Lothag2	GGLN	S/N/N	VLYTLCVERFLV	LQRWFRNRKKMR
162	Plasmb1	GAIT	S/R/N	*Not identified*	IQTQSRILLNR
'HACROBIA'					
Haptophyta					
163	Chryss1	GFLS	S/T/N	ALEAFGKKAFGS	MQNAFRKRLAR
164	Chryss2	ATMT	S/V/N	VLNALVKHNFTS	LQQRFAFEAMR
165	Gephyo1	QLMT	T/I/N	VFDKLALLNASE	LQWIENLQSRR
166	Gephyo2	AMMT	T/A/N	TLTALSQRAFCE	*Not identified*
167	Isochg1	VMVT	A/K/N	VFDRLAQANASE	LSPRFGHNATL
168	Isochg2	AFMT	T/I/N	TLTALAHQATCE	LQSRWRGYAMR
169	Isochg3	GLLT	A/A/S	TLMTLARRNI--	LLAAYRAALVG
170	Isochg4	SLLT	T/S/N	VLTALSYHVSGT	LQQRWRGHMGR
Cryptophyta					
171	Guillt1	PFIT	C/V/N	TFSACVKRVLAI	VQHAYREWKEN
172	Guillt2	PFLT	C/V/N	*Not analysed*	*Not analysed*
173	Guillt3	PFLT	C/Q/N	*Not analysed*	*Not analysed*
174	Guillt4	YTKT	T/A/N	*Not analysed*	*Not analysed*
175	Guillt5	ALLT	A/N/A	MISRTREVTWFA	IFADKRKGRK-
176	Guillt6	xGAS	A/E/S	VMSIIVYWRKPE	*Not identified*
177	Guillt7	AGSS	A/E/N	LVLTMLYWRKPT	*Not identified*
178	Guillt8	ESLQ	T/D/N	VFCIVLKRKLKY	*Not identified*
179	Guillt9	GTSQ	A/S/N	*Deletion*	LQRMYRRWLWR
180	Guillt10	*Not identified*	T/Q/Y	TLGFLQQRDRAP	IQPVMFMRGTR
ARHAEPLASTIDA					
Chlorophyta					
181	Bathyp1	TLTT	V/L/N	Not identified	IQVIIASVRLR
182	Bathyp2	KLLT	S/R/S	CLDALLHAKLGNS	IQRVFRGHRVR
183	Chlamr1	VMLT	T/Y/N	VMHALAGNVCGT	VRAAIKGFLLR
184	Chlamr2	VFVT	T/L/N	TLHALAGRVAGT	VQAAIRGFMAR
185	Chlamr3	AFLT	S/S/N	TAFELVKRCSQA	VQRKWRTRMRA
186	Chlamr4	MLAD	T/N/N	ELKAFAN-----	---ATNLLLTR
187	Chlamr5	AFLT	S/Q/N	TAFELVKRTSQT	*Deletion*
188	Chlamr6	LLVT	T/N/N	*Not identified*	*Not identified*
189	Chlorv1	VMLT	C/T/N	VMQALTARAAGI	IQRAGGQGRGR

№	Name	IG XXXX	DS A/F/N	pre-IQ motif xXxxXX+xxXxX	IQ motif I(L,V)Qxxx+xxxxx+
190	Microp1	GLMT	A/N/N	VLQALTSHAMG-	Not present in seq.
191	Microp2	ILLT	A/Q/N	TLHALAGRIAGT	VQAAVRGFIAR
192	Microp3	NFVT	T/D/N	*Not analysed*	*Not analysed.*
193	Microp4	LFVT	A/Q/N	TIQALAARVDGI	FQAVWRGFIAR
194	Microp5	YSYS	A/D/N	*Not analysed*	*Not analysed*
195	Micros1	VFMT	A/Q/N	TLHALTGRIAGT	VQAAVRGYMQR
196	Micros2	ATMT	A/N/N	ALSAFTTRAMGI	*Not analysed*
197	Micros3	GLMT	A/N/N	*Not analysed*	*Not analysed*
198	Micros4	YCYS	A/D/N	*Deletion*	*Not identified*
199	Micros5	IDLT	S/E/N	TAFELVRRVCEC	VQRHWREVTAA
200	Micros6	VFTT	T/A/N	*Not analysed*	*Not analysed*
201	Micros7	LFVT	A/Q/N	VLKALAARLDGI	VQSAWRR----
202	Micros8	AMAN	C/Y/N	*Not identified*	*Not identified*
203	Monorn1	VLLT	A/K/N	TLHALAGRVAGT	VQAAIKGFLQR
204	Ostrel1	TIMS	A/Q/N	LLQAFTAKAMGV	*Deletion*
205	Ostrel2	KLLT	T/S/S	LLELLATKLGAA	AQSYIRGHITR
206	Ostrel3	TFVT	T/N/N	*Not identified*	*Not identified*
207	Ostret1	TIMS	A/N/N	LLQALTAKAMGV	*Deletion*
208	Ostret2	MILS	T/N/H	*Not analysed*	*Not analysed*
209	Ostret3	KLLT	S/N/S	LLELLATKLGAS	TQAHVRGFIVR
210	Ostret4	PLMT	T/A/N	*Not identified*	LQEAIPSHDTK
211	Ostret5	MILS	T/N/H	*Not analysed*	*Not analysed*
212	Volvoc1	AFLT	T/H/N	TLHALAGSVCAT	*Deletion*
213	Volvoc2	VFMT	A/M/N	TLHALAGRLAGC	*Deletion*
214	Volvoc3	VDLT	A/L/N	VLYALSGAVCGA	VAAAIRGFLLR
215	Volvoc4	AFLT	S/Q/N	TAFELVKRISQT	IQRRWRSRFRA
EXCAVATA					
Euglenozoa, Kinetoplastida					
216	Leishb1	TGTT	T/E/S	VLLPLARRVMAI	LQAAFRRDRAMR
217	Leishi1	TGTT	A/E/S	VLLPLARRVMAI	LQAAFRRDRAMR
218	Leishm1	VGTT	T/E/S	VLLPLARRVMAI	LQAAFRRDHAMR
219	Leptos1	AGTT	T/E/S	VLLPLARRVMAI	LQAAYRRDRAMR
220	Leptos2	ALLT	S/L/S	LLRGLAYR----	*Not identified*
221	Phytosp1	xGMN	T/G/N	VLFSLARRAMRM	IQAAYRRNRAMR
222	Trypab1	LGIT	A/E/N	VLLPLARRVMAI	VQAVCRRKKACR
223	Trypac1	TDIT	T/E/N	VLLPLARRVMAI	VQAAYRRDKACR
224	Trypac2	TDIT	T/E/N	VLLPLARRVMAI	VQAAYRRDKACR
225	Trypag1	MGIT	T/K/N	VLLPLARRVMAI	LQAAYRRDKARR
226	Trypag2	*Not identified*	Q/Y/N	IVQGFAYK----	IQRYWRQRRRAK
227	Trypar1	TDIT	T/E/N	VLLPLARRVMAI	VQAAYRRDKACR
228	Trypav1	LGIT	T/K/N	VLLPLARRVMAI	VQAVCRRKKACR
**OBAZOA**					
APUSOZOA					
Apusomonadida					
229	Thecat1	LMMT	A/I/N	VLLALSRRVNDD	IIEWYSAFKNR
230	Thecat2	VFMT	S/T/N	VLNSLVRFRIAS	*Not identified*
231	Thecat3	VFVT	A/H/A	VVRAFILGEVTH	ISSMARETEAA
232	Thecat4	ALLT	S/Q/N	VFLAKTHARRMA	*Not identified*
233	Thecat5	SLVT	A/K/N	*Not identified*	*Not identified*
234	Thecat6	AFLT	A/S/N	TFEAVIRYAVEH	ISYYWVKYRHV
235	Thecat7	LMMT	A/I/N	VLLALSRRVNDD	IIEWYSAFKNR

№	Name	IG<br>XXXX	DS<br>A/F/N	pre-IQ motif<br>xXxxXX+xxXxX	IQ motif<br>I(L,V)Qxxx+xxxx+
OPISTHOKONTA					
Metazoa (Na_v1)					
236	AplyNav1	MFMT	A/F/N	ILDALTKNFLG-	IQKAYRNFK--
237	BranNav1	LFMT	A/F/N	LLTKLTDKV--	VQRAFRRWMLK
238	DrosNav1	MFMT	A/F/N	ILDALTKDFFAR	IQHAWRKHKAR
239	HaloNav1	IFMT	A/F/N	VLFALTKRVLGE	IQRSWRRYRIR
240	HomoNav1	IFMT	A/F/N	ILFAFTKRVLGE	IQRAYRRHLLK
241	MusmNav1	IFMT	A/F/N	ILFALTKEVLGD	IQRAYRRHLLQ
Metazoa (Na_v2)					
242	BranNav2	IFLT	A/F/N	VLKELTTFTYKR	IQRAFRNHQTA
243	CyanNav	IFLT	S/F/N	VMAALIKRAIGE	IQKVFRRHLLV
244	DrosNav2	MFLT	A/F/N	ILHALVKHVLGH	IQTGWKEYLRR
245	ExaiNav	VLLT	S/F/N	VMQALVRRVLGD	IQRCFRQYLFN
246	HaloNav2	AFLT	A/L/N	VLFALVKRILGE	IQVAWRGFSGI
247	MnemNav	VLLT	A/Y/N	VLQALISRTLGP	LQKALRRFKNY
248	NemaNav2	IFLT	S/F/N	VMQALVRRVIGD	IQKCYRQYRLQ
249	PolyNav	IFLT	A/F/N	VMTALIKRALGE	IQRIFRRHILM
250	StroNav2	MLLS	A/F/N	VLFALTKRVLAD	VQRAYRFFRLR
251	TricNav2	LFMT	A/T/N	VLEALVERVIGQ	IQRAYRHYRLR
Choanoflagellata (Na_v)					
252	MonoNav	LFLT	A/T/N	VLHALIMQVVRQ	LQRHVRKWLEG
Metazoa (HVA Ca_v: Ca_v1)					
253	CyanCavL	CELD	C/W/F	TLFALVRTSLNI	IQEYFRKEKKK
254	DrosCavL	CDLD	C/W/Y	TLFAVVRTSLSI	IQDYFRREKKR
255	HaloCavL	CELD	C/W/Y	TLFALVRTSLKI	IQDYFRKFKES
256	HomoCavL	CELD	C/W/Y	TLFALVRTALRI	IQEYFRKFKKR
257	StylCavL	CELD	C/W/Y	TLFGLIRSSLNI	IQEYFRRFKAR
Metazoa (HVA Ca_v: Ca_v2)					
258	ApisCav2	GEID	C/W/Y	TLFALIRENLNI	ILESWRTTRFG
259	CaenCav2	GDLD	C/W/Y	TLFALIRESLSI	ILENYRARKSG
260	HomoCavN	CSLE	C/W/Y	TLMALIRTALEI	IFDFYKQNKTT
261	HomCavPQ	YSLE	C/W/Y	TLMALIRTALDI	IMEYYRQSKAK
262	HomoCavR	CSLE	C/W/Y	TLMALIRTALDI	IMDYYKQSKVK
263	SchiCav2	LDLD	C/W/Y	TLLALIRESLGI	*Not identified*
Choanoflagellata (HVA Ca_v)					
264	SalpCavL	DGLD	C/W/Y	CLLAIVRNQLHI	LQEIYRENKRQ
Metazoa (LVA Ca_v)					
265	CaenCavT	MREK	T/D/N	*Not identified*	VLEQELIEVER
266	DrosCavT	*Deletion*	T/D/N	*Not identified*	*Not identified*
267	HomoCavT	*Deletion*	T/D/N	*Not identified*	*Not identified*
268	LymnCavT	*Deletion*	T/D/N	*Not identified*	*Not identified*
Metazoa (NALCN)					
269	CaeNALCN	ALLT	E/L/I	VLYMLSY-----	*Not identified*
270	DroNALCN	ALLT	E/L/I	VINMLSY-----	*Not identified*
271	HomNALCN	ALLT	E/L/I	VLSMLSY-----	*Not identified*
272	NemNALCN	ALLT	E/L/T	VLGIIAY-----	*Not identified*
Fungi (Cch)					
273	AspeCch	AYLT	S/K/N	CLMILAHYNVIS	*Not identified*
274	PeniCch	AFLT	S/K/N	CLMILAHYNVIS	*Not identified*
275	PseuCch	AYQT	A/K/S	MMLVLARAKLID	*Not identified*
276	SaccCch	AYFT	L/E/S	LLLQIPLYTAYS	*Not identified*
277	TrichCch	ALLT	V/K/N	MLLMLAQATLIN	*Not identified*

Table S3. Determinants of activity regulation in the analysed sequences of four-domain voltage-gated cation channels.

IG – inactivation gates; DS – docking site; H – histidine (may be positively charged); X – hydrophobic amino acid residue; X – polar amino acid residue; X – lysine (K) or arginine (R); X – charged amino acid residue; X – conservative residue in the docking site; x – any amino acid residue. *Not analysed* - motif unidentified due to incomplete sequence. *Not identified* – motif unidentified due to low sequence homology of the respective region.

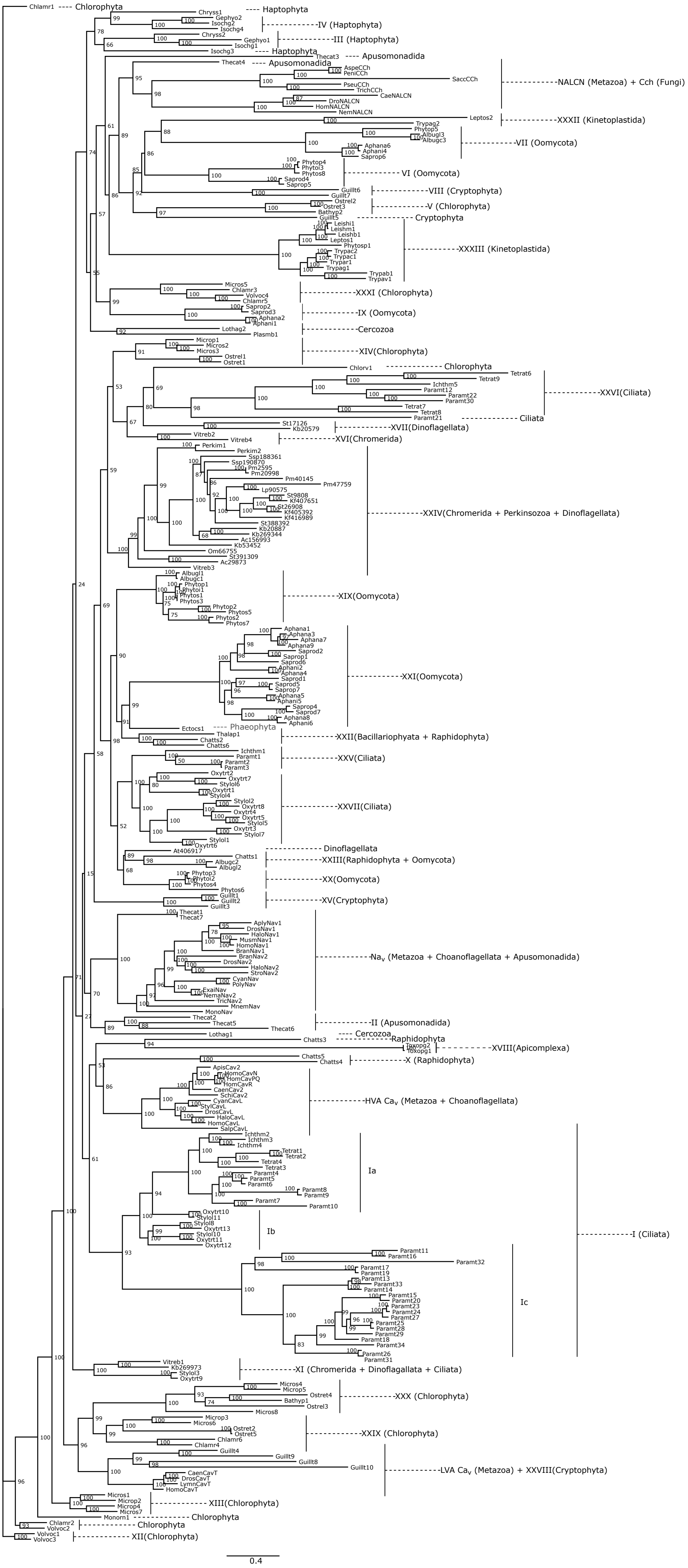
Chlorophyta
Haptophyta
IV (Haptophyta)
III (Haptophyta)
Haptophyta
Apusomonadida
Apusomonadida
NALCN (Metazoa) + Cch (Fungi)
XXXII (Kinetoplastida)
VII (Oomycota)
VI (Oomycota)
VIII (Cryptophyta)
V (Chlorophyta)
Cryptophyta
XXXIII (Kinetoplastida)
XXXI (Chlorophyta)
IX (Oomycota)
Cercozoa
XIV(Chlorophyta)
Chlorophyta
XXVI(Ciliata)
Ciliata
XVII(Dinoflagellata)
XVI(Chromerida)
XXIV(Chromerida + Perkinsozoa + Dinoflagellata)
XIX(Oomycota)
XXI(Oomycota)
Phaeophyta
XXII(Bacillariophyta + Raphidophyta)
XXV(Ciliata)
XXVII(Ciliata)
Dinoflagellata
XXIII(Raphidophyta + Oomycota)
XX(Oomycota)
XV(Cryptophyta)
Na$_v$ (Metazoa + Choanoflagellata + Apusomonadida)
II (Apusomonadida)
Cercozoa
Raphidophyta
XVIII(Apicomplexa)
X (Raphidophyta)
HVA Ca$_v$ (Metazoa + Choanoflagellata)
Ia
Ib
I (Ciliata)
Ic
XI (Chromerida + Dinoflagellata + Ciliata)
XXX (Chlorophyta)
XXIX (Chlorophyta)
LVA Ca$_v$ (Metazoa) + XXVIII(Cryptophyta)
XIII(Chlorophyta)
Chlorophyta
Chlorophyta
XII(Chlorophyta)
0.4

Figure S1. Maximal-likelihood phylogenetic tree of eukaryotic four-domain voltage-gated cation channels obtained by means of IQ-tree. Designation of WSCs corresponds to that on Figures 2 and 3. Bootstrap values are shown.
